# Design and Biological Evaluation of ALKBH2 and ALKBH5 Inhibitors as Adjuvants to Temozolomide-Based Glioblastoma Treatment

**DOI:** 10.3390/biology15161371

**Published:** 2026-08-12

**Authors:** Mirko Rivara, Alessio Malacrida, Martina Ghizzi, Angela Bentivegna, Francesco Saverio Sica, Francesca Re, Stefano Motta, Lara Callea, Laura Bonati, Matteo Incerti, Valentina Zuliani, Gabriella Nicolini

**Affiliations:** 1 Food and Drug Department, University of Parma, Parco Area delle Scienze 27/A, 43124 Parma, Italy; mirko.rivara@unipr.it (M.R.); matteo.incerti@unipr.it (M.I.); valentina.zuliani@unipr.it (V.Z.); 2 Experimental Neurology Unit, School of Medicine and Surgery, University of Milano-Bicocca, Via Cadore 48, 20900 Monza, Italy; gabriella.nicolini@unimib.it; 3 School of Medicine and Surgery, University of Milano-Bicocca, Via Cadore 48, 20900 Monza, Italy; m.ghizzi@campus.unimib.it (M.G.); angela.bentivegna@unimib.it (A.B.); francesco.sica@unimib.it (F.S.S.); francesca.re1@unimib.it (F.R.); 4 PhD Program in Neuroscience, School of Medicine and Surgery, University of Milano-Bicocca, 20900 Monza, Italy; 5 Fondazione IRCCS San Gerardo dei Tintori, 20900 Monza, Italy; 6 GBM-BI-TRACE (GlioBlastoMa-BIcocca-TRAnslational-CEnter), University of Milano-Bicocca, 20900 Monza, Italy; 7 Inter-University Center for the Promotion of the 3Rs Principles in Teaching & Research (Centro 3R), 56122 Pisa, Italy; stefano.motta@unimib.it; 8 Department of Earth and Environmental Sciences, University of Milano-Bicocca, Piazza della Scienza 1, 20126 Milan, Italy; lara.callea@unimib.it (L.C.); laura.bonati@unimib.it (L.B.)

**Keywords:** glioblastoma, temozolomide, drug resistance, patient-derived glioma stem cells, ALKBH

## Abstract

This study reports the design, synthesis, and biological evaluation of novel inhibitors targeting the epigenetic enzymes ALKBH2 and ALKBH5 as potential adjuvants to temozolomide therapy in glioblastoma. Given their critical role in DNA/RNA demethylation, tumor progression, and drug resistance, their inhibition represents a promising therapeutic strategy. Building on the previously identified lead compound MV1035, we employed structure-based drug design to develop new derivatives, including a second-generation series incorporating a fumarate hydrazide moiety to enhance binding affinity through interaction with both substrate- and cofactor-binding sites. Molecular docking studies predicted significantly improved binding for a set of new compounds but, due to multiple synthetic drawbacks, only a subset of the designed series was synthesized and evaluated biologically. MV3030 emerged as the most promising candidate. MV3030 demonstrated an inhibitory effect on ALKBH2 comparable to MV1035, also showing a more moderate inhibitory effect on ALKBH5. Notably, it exhibited intrinsic cytotoxicity in U87-MG cells and patient-derived glioma stem cells, whereas normal astrocytes exhibited markedly higher resistance to the treatment. Furthermore, MV3030 enhanced temozolomide efficacy and displayed favorable blood–brain barrier permeability both in silico and in vitro. Moreover, MV3030 modulated the FoxM1/Wnt/β-catenin axis. Overall, these findings identify MV3030 as a promising compound with the potential to overcome temozolomide resistance and improve glioblastoma treatment.

## Simple Summary

Glioblastoma is a highly aggressive brain tumor that remains extremely difficult to treat because it quickly develops resistance to standard chemotherapy drugs like temozolomide. This study aims to design and evaluate new molecules capable of blocking specific enzymes that drive tumor progression and drug resistance, thereby making standard treatments more effective. Through computer-aided design and laboratory testing, we identified a promising new molecule named MV3030. This molecule demonstrated a strong ability to cross the protective blood–brain barrier and directly reduce the survival and self-renewal of both standard and highly aggressive patient-derived brain tumor cells. Crucially, when combined with the standard chemotherapy drug temozolomide, MV3030 successfully modulated the tumor’s survival signals and significantly enhanced the drug’s ability to kill cancer cells. Our study concludes that MV3030 is an effective tool to overcome drug resistance and halt tumor growth.

## 1. Introduction

Glioblastoma is among the most aggressive primary tumors of the central nervous system, with a poor prognosis and a median overall survival of approximately 14–16 months despite maximal treatment. Standard treatments such as surgery and temozolomide-based chemotherapy remain largely insufficient to achieve long-term disease control. Recent biotechnological advances have enabled the development of more precise therapeutic strategies, such as targeted molecular inhibitors, advanced radiation techniques, and innovative immunotherapies, including CAR-T cells [1,2], many of which are still under investigation.

Increasing evidence indicates that epigenetic dysregulation plays an important role in glioblastoma biology, contributing to tumor progression, therapy resistance, and pronounced intratumoral heterogeneity [3].

Among epigenetic regulators, members of the AlkB family act as Fe(II)/α-ketoglutarate-dependent dioxygenases that catalyze the dealkylation of DNA and RNA nucleobases, thereby influencing diverse biological functions ranging from genomic DNA repair to the epigenetic modulation of nucleic acids [4].

In our previous studies, we identified the imidazobenzoxazin-5-thione derivative, MV1035, as a potent dual inhibitor of the RNA demethylase ALKBH5 and DNA demethylase ALKBH2 [5,6]. By targeting ALKBH5, MV1035 significantly impairs the migratory and invasive capabilities of U87-MG glioblastoma cells. Furthermore, because MV1035 inhibits ALKBH2, a key mediator of temozolomide (TMZ) resistance, its co-administration with TMZ results in a synergistic reduction in cell viability and neurosphere-forming ability in both U87-MG and patient-derived (PD) glioma stem cells (GSC). These findings highlight the MV1035-based approach as a promising strategy to simultaneously target tumor aggressiveness and overcome TMZ resistance in glioblastoma.

In a more recent work [7], we synthesized and biologically evaluated a series of analogs derived from the lead compound MV1035, in order to explore structure–activity relationships and improve target engagement. These results indicated that opening the tricyclic scaffold while maintaining the imidazole ring preserved affinity for both ALKBH2 and ALKBH5 [5,6,7].

**Figure 1:**
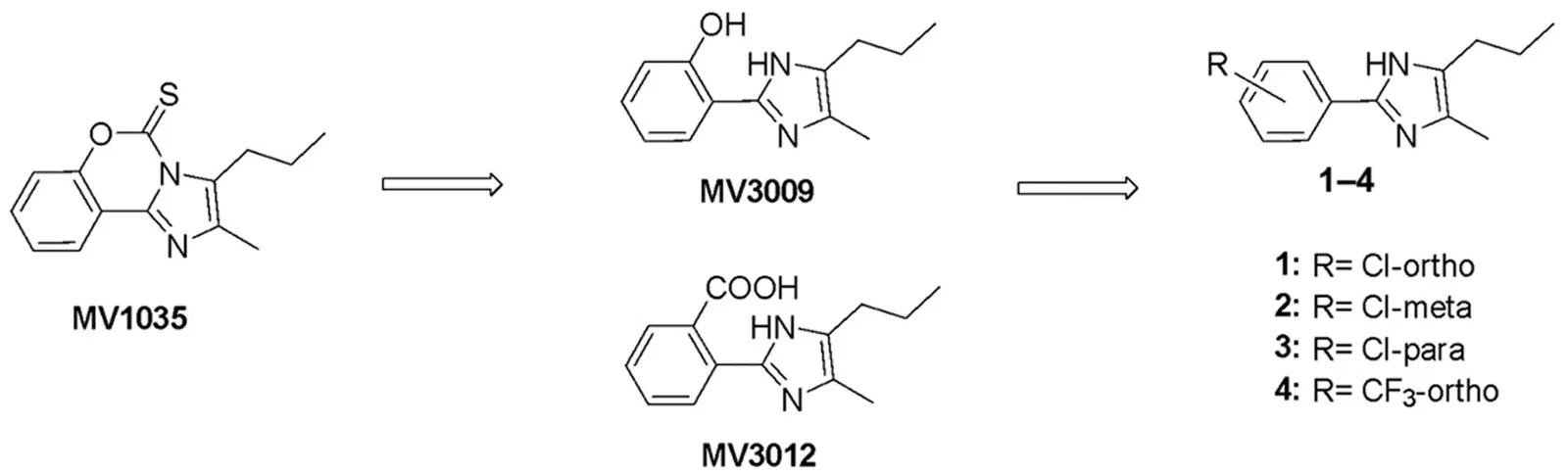
First set of compounds, derived from MV3009 and MV3012 bearing different substituents on the phenyl ring.

**Figure 2:**
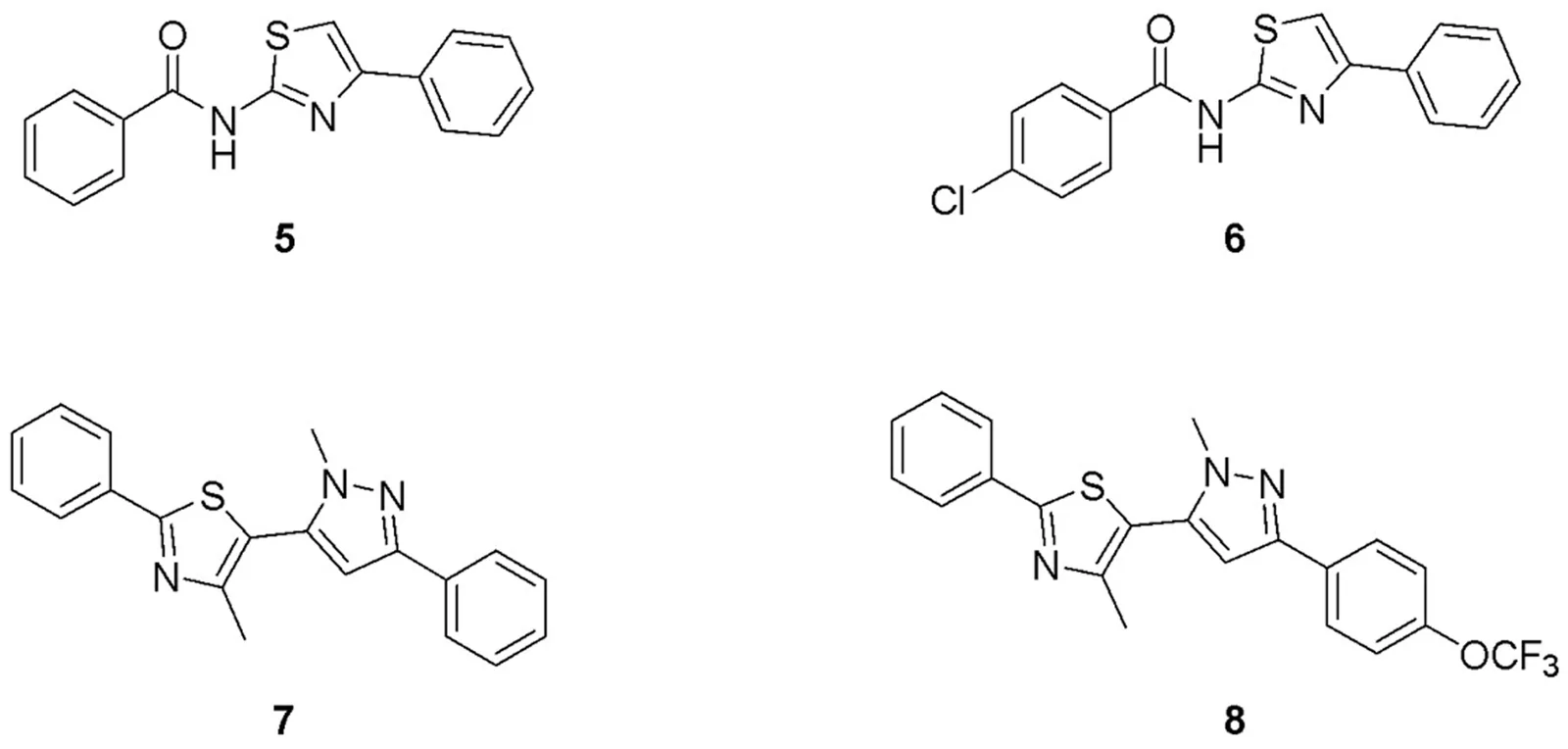
Compounds synthesized following a scaffold-hopping strategy.

Based on these findings, we initially focused on further substitutions on the phenyl ring by inserting a chlorine atom and a trifluoromethyl group (Figure 1). In parallel, we pursued a scaffold-hopping strategy (Figure 2) aimed at replacing the imidazole ring with different heterocycles, to assess their impact on enzyme inhibition and cellular activity.

As we expanded the chemical space and explored additional scaffold options to further advance the project, we recognized the need for a deeper understanding of how the reported compounds interact with their target enzymes. In particular, elucidating their putative binding modes became a key prerequisite for guiding further rational optimization. To address this, we initiated a structure-based drug design campaign. We investigated the binding modes and key interactions of the previously published compounds (MV3003–MV3012) and the newly synthesized ones (**18**; Table 1) performing molecular docking studies using the ALKBH2 and ALKBH5 structures. The primary goal of this initial computational step was to understand the baseline interactions of these nitrogen-containing heterocyclic scaffolds within the enzymatic active sites. Building upon the structural insights derived from this first series, we rationally designed a second generation of derivatives aimed at expanding the interaction network within the catalytic pockets to improve binding affinity and dual-target engagement. In this work, we report the design, synthesis, and comprehensive biological evaluation of these novel ALKBH2/ALKBH5 inhibitors. The selected candidates were subjected to enzymatic assays and in vitro blood–brain barrier permeability evaluations. Furthermore, the most promising compound was thoroughly characterized by its intrinsic cytotoxicity and its synergistic potential when combined with temozolomide in both U87-MG glioblastoma cells and patient-derived glioma stem cells, and we ultimately investigated its mechanistic impact on the FoxM1/Wnt/β-catenin signaling axis.

## 2. Materials and Methods

### 2.1. Molecular Docking

The crystal structure of the human ALKBH2 enzyme in complex with double-stranded DNA (PDB ID: 3RZK) [8] and the human ALKBH5 enzyme in complex with single-stranded RNA (PDB ID: 7WKV) [9] were retrieved from the Protein Data Bank. Prior to docking, the protein structure was processed using Schrödinger’s Protein Preparation Wizard (Schrödinger Suite 2024-2) [10]. To standardize the receptor cavities for docking, all crystallographic water molecules were strictly removed from the structures. Hydrogen atoms were added, missing side chains were built, and bond orders were assigned. The protonation states of ionizable residues were predicted using PROPKA [11] at pH 7.0. The prepared protein–metal complexes were finally subjected to restrained minimization using the OPLS4 force field to relieve steric clashes while maintaining heavy-atom coordinates.

Ligand structures were built de novo using Maestro’s Builder tool. To accurately model the metal coordination driven by the fumarate hydrazide moiety, preliminary protocol validation and calibration were performed using the homologous FTO enzyme co-crystallized with a structurally related acylhydrazine inhibitor (PDB ID: 4CXY). Analysis of the 4CXY complex, carried out using the Protein Preparation Wizard, indicated a strong preference for a deprotonated (negatively charged) state at the hydrazide nitrogen involved in metal coordination. Subsequent redocking validation experiments of the native ligand into the FTO active site confirmed that the experimental crystallographic binding pose could only be accurately reproduced when this specific deprotonated state was explicitly assigned. Based on this structural validation, we concluded that this class of linkers effectively coordinates the catalytic metal in a deprotonated form. Consequently, this specific protonation state was manually assigned to all designed compounds sharing the fumarate hydrazide linker. The ligands were then processed using the LigPrep module (Schrödinger Suite 2024-2) to generate low-energy 3D conformers, allowing the generation of up to a maximum of 32 stereoisomers per ligand. Other general protonation states were assigned at pH 7.0 ± 0.5 using the Epik Classic module [12].

Docking calculations were performed using the Glide XP (extra precision) algorithm [13]. The receptor grids for both ALKBH2 and ALKBH5 were generated without applying any artificial spatial or metal-coordination constraints, allowing the pre-calibrated negatively charged ligands to freely guide the interaction geometry. The center of the receptor grid was defined by calculating the center of mass of the active site residues lining the binding cavity, which structurally positioned the grid center immediately adjacent to the catalytic metal ion. To ensure comprehensive coverage of the extended substrate-binding tunnel (encompassing both the methylated base-binding pocket and the cofactor-binding site), the bounding box parameters were set with an inner box (defining the allowable space for the ligand center) of 12 × 12 × 12 Å, and an outer box (defining the absolute spatial limit for all ligand atoms) of 25 × 25 × 25 Å. Ligands were docked flexibly, with full conformational sampling of the rings and nitrogen inversions.

### 2.2. In Silico Prediction of Endothelial Permeability of MV1035 and Its Derivatives

To facilitate the rapid and cost-effective screening of anti-glioblastoma drug candidates, reliable indicators of blood–brain barrier (BBB) permeability are essential. This is particularly important for efficiently screening large numbers of compounds and prioritizing the most promising candidates for further evaluation. Therefore, we applied the “Central Nervous System—Multiparameter Optimization” (CNS-MPO) approach [14]. The algorithm evaluates six fundamental physicochemical properties—clogP, clogD, molecular weight (MW), topological polar surface area (TPSA), number of hydrogen bond donors (HBDs), and pKa—assigning scores on a scale from 0 to 6.0. Compounds achieving a CNS-MPO score of 4.0 or higher are generally considered strong candidates for central nervous system (CNS) penetration [15]. Temozolomide has been used as reference (Sigma-Aldrich, St. Louis, MO, USA).

### 2.3. ALKBH2 Cell-Free Activity Assay

The DNA demethylation activity of recombinant ALKBH2 was assessed using a cell-free biochemical assay. Briefly, 0.6 µg of active recombinant ALKBH2 protein (Active Motif, Carlsbad, CA, USA) was incubated with 1 µg of a methylated single-stranded DNA (ssDNA) substrate (5′-AAAGCAG(1-mA)ATTCGAAAAAGCGAAA-3′; Ella Biotech, Fürstenfeldbruck, Germany). The reactions were performed in a final volume of 50 µL of reaction buffer (50 mM HEPES pH 8.0, 50 µM Fe(NH_4_)_2_(SO_4_)_2_, 1 mM α-Ketoglutarate (α-KG) and 2 mM ascorbic acid) in the presence or absence of compounds **1**–**15**. After incubation at 37 °C for 30 min, the enzyme was thermally inactivated at 95 °C for 5 min. The resulting ssDNA was hybridized with an equimolar amount of a complementary unmethylated sequence (3′-TTTCGTCTTAAGCTTTTTCGCTTT-5′). Annealing was conducted in a thermal cycler using the following program: 95 °C for 2 min, 25 °C for 45 min, 4 °C for 5 min. The annealed double-stranded DNA (dsDNA) was subsequently digested with EcoRI (Thermo Fisher Scientific, Waltham, MA, USA) at 37 °C for 45 min, according to the manufacturer’s instructions. The reaction was terminated by heat inactivation at 65 °C for 20 min. Digested and undigested DNA fragments were resolved by native polyacrylamide gel electrophoresis. Gels were stained with SYBR Safe (Thermo Fisher Scientific, Waltham, MA, USA) and visualized using an Amersham Imager 600 (GE Healthcare, Chicago, IL, USA).

### 2.4. ALKBH5 Cell-Free Activity Assay

The RNA demethylation activity of ALKBH5 was evaluated using a cell-free biochemical assay. Briefly, 4 μM of active recombinant ALKBH5 protein (Active Motif, Carlsbad, CA, USA) was incubated with 80 μM of M6A-ssRNA (GG-m6A-CU; Ella Biotech, Fürstenfeldbruck, Germany). The reaction was performed in a final volume of 50 µL containing: 50 mM HEPES buffer (pH 7.5), 150 μM α-KG, 150 μM Fe(NH_4_)_2_(SO_4_)_2_, and 2 mM ascorbic acid (all from Sigma-Aldrich, St. Louis, MO, USA). The mixture was incubated at 4 °C for 30 min in the presence or absence of compounds **1**–**15**. To quantify the remaining methylation levels, 5 μL of each reaction mixture was spotted onto a nylon membrane. m6A levels were detected via dot blot analysis according to the manufacturer’s instructions. The membrane was probed with a primary anti-m6A-RNA antibody (1:250; Sigma-Aldrich, St. Louis, MO, USA) followed by a secondary anti-mouse antibody (1:2000; Jackson Immuno Research, Ely, UK). Chemiluminescent signals were visualized using an Amersham Imager 600 (GE HealthCare, Chicago, IL, USA).

### 2.5. In Vitro Model of the Blood–Brain Barrier (BBB)

The in vitro BBB model was established using hCMEC/D3 cells (5.6 × 10^4^ cells) seeded on collagen-coated transwell inserts (polyester 12-well, pore size 0.4 μm, translucent membrane inserts 1.12 cm^2^; Costar, Kennebunk, ME, USA) to form a polarized monolayer. After 2 days of growth, the basal medium was replaced with EBM-2 supplemented with 5% FBS, 1% chemically defined lipid concentrate, 1% P/S, 10 mM HEPES, 5 μg mL^−1^ ascorbic acid, 1.4 μM hydrocortisone, and 10 mM LiCl, to promote barrier properties (all from Euroclone S.p.A., Milan, Italy) [16]. Monolayer integrity was assessed by transendothelial electrical resistance (TEER) measurements, and by testing permeability to TRITC-dextrans (4 kDa). On day 7, when TEER peaked and permeability was lowest, compounds were dissolved in PBS, and 3% DMSO was added to the apical side (concentration ranging from 25 to 100 µM) and incubated at 37 °C for up to 3 h. The amount of compound in the basolateral side was measured over time using a NanoDrop One C (Thermo Scientific, Waltham, MA, USA). The effect of compounds on hCMEC/D3 cell viability was assessed by MTT assay [17].

### 2.6. Cell Culture Conditions

We used the U87-MG cell line and two patient-derived glioma stem cell lines (PD-GSCs), G179 [18] and GSC7 [19]. Both PD-GSC lines were established from primary glioblastoma specimens of male donors and maintained under serum-free conditions to preserve their stemness and tumor-specific phenotypes.

G179 isolation and characterization is reported in Pollard’s work [18]. GSC7 isolation and characterization is reported in Giambra’s work [19]. G179 and GSC7 have been authenticated using Short Tandem Repeat profiling within the last three years (Supplementary Table S2, [20]).

In line with current WHO criteria for adult glioblastoma, both PD-GSC lines are IDH-wildtype. Regarding MGMT promoter status, G179 cells display MGMT promoter hypermethylation (conferring higher baseline sensitivity to temozolomide), whereas GSC7 cells are MGMT unmethylated, representing an MGMT-driven chemoresistant phenotype [18,19].

The U87-MG cell line was cultured in DMEM low-glucose medium, supplemented with 10% FBS, 1% L-glutamine and 1% penicillin and streptomycin (Euroclone S.p.A., Milan, Italy).

PD-GSCs were cultured in a selective medium for NSC composed of DMEM F-12 and Neurobasal 1:1; B-27 supplement without vitamin A (Life Technologies Italia, Milan, Italy); 2 mM L-glutamine (Euroclone S.p.A., Milan, Italy); 10 ng/mL recombinant human bFGF and 20 ng/mL recombinant human EGF (Miltenyi Biotec, Bergisch, Gladbach, Germany); and 20 UI/mL penicillin and 20 μg/mL streptomycin (Euroclone S.p.A., Milan, Italy).

Immortalized human astrocytes were employed as a non-cancerous control model to evaluate compound toxicity in healthy cells. The cells were maintained in astrocyte medium supplemented with 2% fetal bovine serum, 1% astrocyte growth supplement and 1% penicillin/streptomycin (Innoprot, Derio, Bizkaia, Spain).

Cells were incubated at 37 °C and 5% CO_2_ in a humidified incubator.

### 2.7. MTT Assay

The viability of U87-MG, G179 and GSC7 cells was evaluated using the MTT [3-(4,5-dimethylthiazol-2-yl)-2,5-diphenyltetrazolium bromide] assay (Sigma-Aldrich, St. Louis, MO, USA). Cells were seeded in 96-well plates at a density of 5 × 10^3^ cells/well and allowed to adhere overnight. All experimental conditions were performed in quadruplicate (four wells per condition). Cells were treated with the synthesized compounds and/or TMZ using different experimental setups: for the initial screening, U87-MG cells alone were exposed to compounds **1**–**15** at a fixed concentration of 50 µM for 24, 48, and 72 h. Subsequently, all three cell lines were treated for 24 h with combinations of MV3030 (0, 10, 25, and 50 µM) and TMZ (0, 300, 600, and 1200 µM). After treatment, an MTT solution was added directly to the culture medium to reach a final concentration of 0.5 mg/mL. After a 4 h incubation period at 37 °C, the plates were centrifuged at 1000× *g* for 10 min to ensure the sedimentation of formazan crystals. The supernatant was carefully aspirated and formazan precipitates were dissolved in acidified 2-propanol. The absorbance was measured at 570 nm using a microplate reader (BMG LABTECH, Ortenberg, Germany). Cell viability was expressed as a percentage relative to the untreated control.

### 2.8. Limiting Dilution Assay

PD-GSCs were seeded as single-cell suspensions in 96-well plates at specific densities adjusted according to the proliferation rate of each line. Specifically, GSC7 cells were seeded at 800, 1600, and 3200 cells/well, while G179 cells were seeded at 300, 600, 1200 cells/well, using eight technical replicates per condition. Treatments were performed on day 0 immediately after seeding with DMSO (control), 600 µM TMZ, 50 µM MV1035, 50 µM MV3030, or their respective combinations. At this time, wells containing pre-existing cell aggregates were identified via optical microscopy (Eclipse TS100; Nikon Corporation, Tokyo, Japan) and excluded from subsequent analysis to guarantee clonal evaluation. After 7 days of incubation, the formation of neurospheres was evaluated, and wells containing cellular aggregates composed of at least 4 cells were scored as positive for sphere formation. Stem cell frequencies and statistical significance between groups were calculated via the single-hit Poisson model using the Extreme Limiting Dilution Analysis (ELDA) software (https://bioinf.wehi.edu.au/software/elda/, accessed on 1 July 2026) [21]. Pairwise comparisons between groups were evaluated using the log-likelihood ratio test (Chi-square statistics) built into the ELDA platform. The assumption of the single-hit model was validated using the log-likelihood ratio test provided by the software, and no arbitrary outlier exclusions were applied.

### 2.9. Western Blotting

Protein expression was evaluated by Western blotting. First, 250 × 10^3^ U87-MG, G179, and GSC7 cells were seeded and treated with MV3030 (or MV1035) 50 µM, TMZ 600 µM and their combination. After 24 h, cells were chemically and mechanically lysed with RIPA buffer and a cell scraper. Protein samples were then clarified by centrifugation (13000× *g*, 15 min, 4 °C) and quantified using the Bradford method. Then, 10 µg of proteins were separated into an SDS-PAGE gel and transferred to a nitrocellulose membrane. Western blotting was performed following the antibody manufacturer’s instructions (anti-FoxM1 (1:1000), anti-β-Catenin (1:1000), anti-Survivin (1:1000), anti-Cyclin E (1:1000), and anti-Actin (1:1000), Cell Signaling Technology, Danvers, MA, USA; anti-Vinculin (1:1000), Santa Cruz Biotechnology, Dallas, TX, USA; anti-Mouse IgG HRP-labeled (1:2000), Chemicon, Temecula, CA, USA; anti-Rabbit IgG HRP-labeled (1:2000), Perkin Elmer, Waltham, MA, USA). Band detection was performed using an Azure Imager 600 (Azure Biosystems, Dublin, CA, USA) and quantification was performed using ImageJ (version 1.54k).

### 2.10. Immunofluorescence

Protein subcellular localization was evaluated by immunofluorescence. Briefly, 250 × 10^3^ U87-MG, G179, and GSC7 cells were seeded and treated with MV3030 (or MV1035) 50 µM, TMZ 600 µM and their combination. After 24 h, cells were fixed with 10% neutral buffered formalin for 10 min and subsequently incubated with 0.3 M glycine for 15 min. Following fixation, cells were blocked and permeabilized for 1 h at room temperature using a solution containing 3% BSA, 10% normal donkey serum and 0.1% Triton X-100 (all from Sigma-Aldrich, St. Louis, MO, USA). Cells were then incubated overnight at 4 °C with primary antibodies against FoxM1 (1:100) and β-Catenin (1:200). Subsequently, cells were incubated with fluorochrome-conjugated secondary anti-rabbit (1:250, Alexa Fluor 488) and anti-mouse (1:250, Alexa Fluor 555) antibodies (Thermo Fisher Scientific, Waltham, MA, USA) for 1 h. Finally, coverslips were mounted using an aqueous mounting medium containing DAPI. Images were acquired using a Zeiss LSM 710 Confocal Microscope (Carl Zeiss, Oberkochen, Germany).

### 2.11. Statistical Analysis

All experiments were performed at least in triplicate, and data are presented as mean ± standard deviation (SD). Except for the limiting dilution and drug-interaction modeling, statistical significance was evaluated using one-way ANOVA followed by Tukey’s post hoc test for experiments that satisfied the assumptions of normality (Shapiro–Wilk test) and homogeneity of variance (Levene’s test). For the experiments in Figures 14 and 15, due to a violation of the normality and homogeneity assumptions, the non-parametric Kruskal–Wallis test followed by Dunn’s test was applied. Statistical analyses and graphical representations were generated using GraphPad Prism software (version 8, San Diego, CA, USA). *p*-values less than 0.05 were considered statistically significant across all analyses.

Synergistic effects between MV3030/MV1035 and TMZ were evaluated using the Zero Interaction Potency (ZIP) and the Bliss independence models. Synergy scores were calculated: a synergy score greater than 10 was considered significant and indicative of a synergistic interaction, while scores between −10 and 10 suggested an additive effect, and scores less than −10 indicated antagonism. Synergistic, additive, or antagonistic interactions between TMZ and MV3030 were also quantitatively assessed using the Chou–Talalay method, based on the median-effect equation. The Combination Index (CI) was calculated for each drug combination. Drug interactions were classified based on the resulting CI values, where CI < 1 indicates synergy, CI = 1 additivity, and CI > 1 antagonism. Drug combination synergy analyses were performed in R software (version 4.6.0) using the SynergyFinder package.

To assess statistically significant differences in drug responsiveness, cell viability curves of the three glioblastoma cell lines were compared against non-tumoral astrocytes using the Extra sum-of-squares F-test. Statistical significance was set at *p* < 0.05. Overall differences in synergy between cell lines were evaluated by calculating the delta synergy score (Δ Synergy Score), defined as the difference between the respective synergy scores of glioblastoma lines and non-tumoral astrocytes.

## 3. Results

### 3.1. Molecular Docking Studies

**Figure 3:**
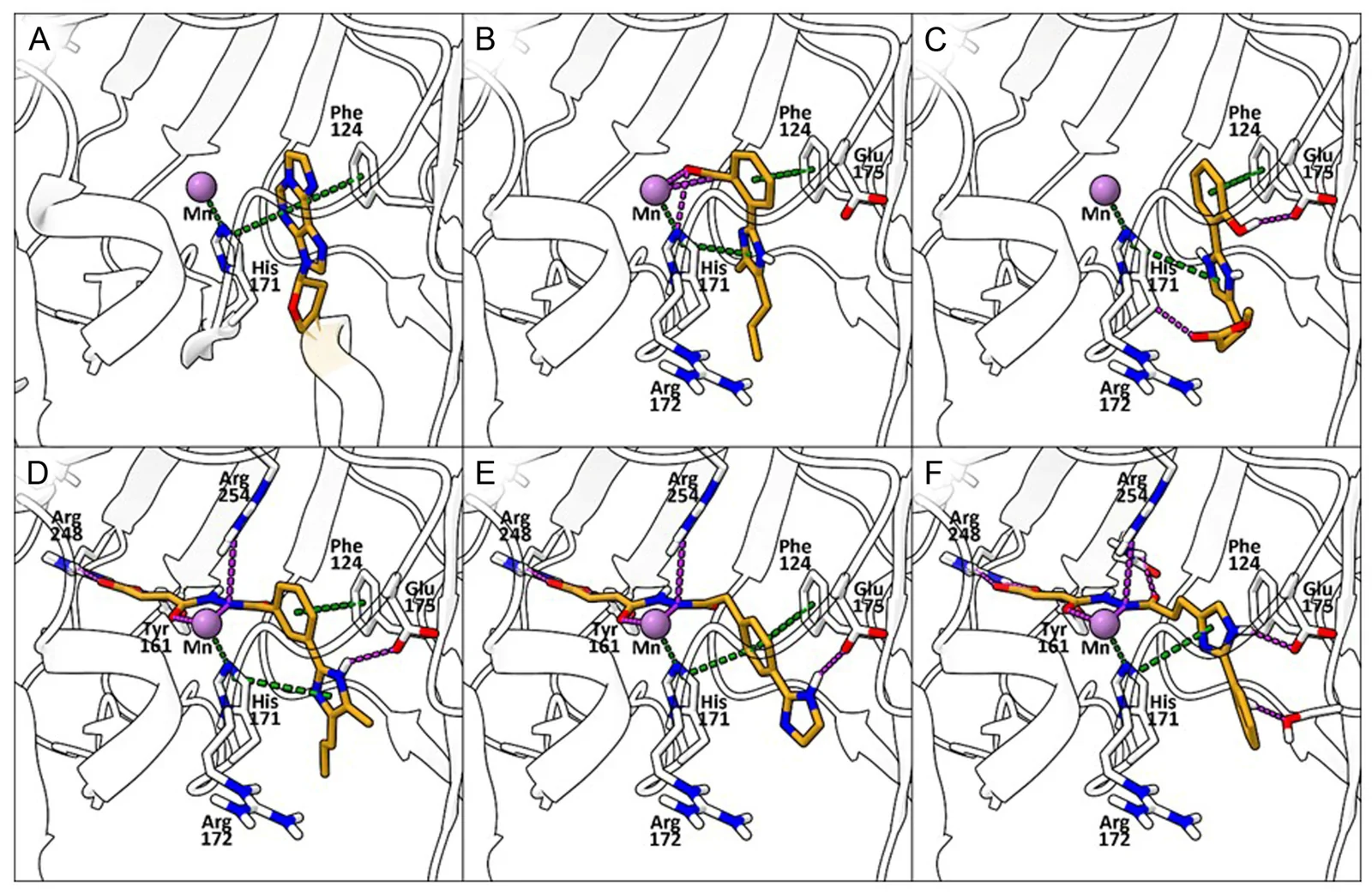
Comparison of binding modes within the ALKBH2 active site (derived from PDB ID: 3RZK). Crystallographic binding mode of the DNA base substrate (**A**). Predicted binding pose obtained from molecular docking for compounds: MV3012 (**B**); MV3006 (**C**); **T4** (**D**); **T3** (**E**); **T7** (**F**). Key interacting residues are shown as stick models.

We first investigated the binding modes and key interactions of the previously published compounds (MV3003–MV3012) and the newly synthesized ones (**1**–**8**; Table 1), performing molecular docking studies using both ALKBH2 and ALKBH5 structures. These compounds feature nitrogen-containing heterocyclic scaffolds structurally reminiscent of nitrogen bases and were predicted to primarily occupy the DNA base-binding region of the active site. Their binding modes often mimicked that of the natural DNA substrate (Figure 3A–C), suggesting a mechanism of competitive inhibition. Notably, compound MV3012 exhibited the most favorable docking score within this initial series (Supplementary Table S1), which can be attributed to its carboxylic acid moiety coordinating the catalytic Mn(II) ion and anchoring the ligand within the active site (Figure 3B). Despite these favorable interactions, the overall docking scores obtained for the series were moderate (Supplementary Table S1), indicating that further structural optimization would be useful to improve binding affinity.

Based on these findings, we pursued a rational optimization strategy aimed at enhancing binding affinity by extending ligand interactions into the adjacent cofactor-binding cavity of ALKBH2. Taking inspiration from successful inhibitor development efforts targeting the related FTO protein (also known as ALKBH9) [22], we hypothesized that the introduction of a moiety capable of mimicking key α-KG interactions could significantly improve target engagement. In particular, in FTO inhibitors, a “fumarate hydrazide” chain was particularly effective, chelating the metal ion and forming key contacts within the cofactor-binding pocket.

We thus selected a phenyl-imidazole scaffold, derived from MV3006, one of the most active compounds tested by us, as a synthetically tractable and promising starting point for this second design cycle. A new series of compounds (**T1**–**T7**; Table 2) was designed by incorporating a fumarate hydrazide chain onto a phenyl-imidazole core. Different substitution patterns and attachment points were explored, including meta (**T1**–**T3**)- and para (**T4**)-substitution on the phenyl ring; reintroduction of methyl and propyl substituents on the imidazole ring (**T3**); attaching the hydrazide chain to the imidazole ring instead of the phenyl ring (**T5**); and introduction of ortho-substituents such as hydroxyl (**T6**) or chlorine (**T7**) on the phenyl ring of the imidazole-linked analogs.

Molecular docking calculations performed on this second generation of fumarate hydrazide derivatives (**T1**–**T7**) revealed a substantial improvement in predicted binding affinities, as reflected by markedly enhanced docking scores (Table 2). The predicted binding poses confirmed successful engagement of the fumarate hydrazide moiety within the cofactor-binding pocket (Figure 3D–F). In these poses, the deprotonated hydrazide group chelates the Mn(II) ion, mimicking the metal-coordination pattern of α-KG. This interaction is predicted to be further stabilized by electrostatic interactions with the conserved residue Arg254. Additionally, the fumarate moiety extends into the cavity, enabling the formation of a salt bridge between its terminal carboxylate and Arg248. The aromatic portions of the molecules remain anchored in the DNA base-binding region, maintaining key interactions previously observed in the initial compound set.

Among the new series, **T3**, **T4**, and **T6** exhibited the most promising docking scores and binding geometries (Figure 3D), displaying a high degree of structural congruence with previously reported fumarate-containing FTO inhibitors [22].

These findings suggest that introducing a fumarate hydrazide chain could successfully extend the interaction network of the parent scaffold, contributing to enhanced predicted affinity through dual engagement of both the substrate- and cofactor-binding cavities. We also evaluated the putative interaction of the **T1**–**T7** series with the ALKBH5 isoform. Molecular docking calculations yielded high theoretical affinity scores for ALKBH5, which were generally comparable to those obtained for ALKBH2 (Table 2). These scores suggest that the fumarate hydrazide derivatives can theoretically fit within the active sites of both enzyme isoforms.

### 3.2. Synthesis

The synthesis of key intermediates and final compounds along with the yields, purity and spectroscopic characterization of the compounds tested are reported in the Supporting Information. As further discussed below, not all the target compounds (**T1**–**T7**) were synthesized due to drawbacks both in the first step of the synthesis as well as during the final saponification reaction. Table 3 reports the compounds synthesized and tested, which partially differ from those designed and presented in Table 2. They represent a compromise between synthetic accessibility and the virtually proposed structures, while consistently preserving the key structural features deemed essential for maintaining binding affinity.

Synthesis of compounds **1**–**4** was performed following well-known techniques for preparing 2 phenyl imidazole [23]; briefly, the substituted aldehyde was reacted with the hexane-2,3-dione in methanol using ammonium chloride as the source of ammonia. The reactions were performed at room temperature (RT), leading, after purification through flash chromatography, to good yields of the desired compounds.

Compounds **5** and **6** were obtained from the reaction between 4-phenylthiazol-2-amine and benzoylchloride or 4-chloro benzoylchloride, leading to compounds **5** and **6,** respectively, in good yields [24].

Compounds **7** and **8** were obtained starting from 2-phenylthiazole chalcones following ring closure with methyl hydrazine.

Compounds **9**–**15** were synthesized following a modification of the methodology already published in [25].

The key steps in making compounds **9**–**11** were the synthesis of imidazole intermediates followed by a reaction with the monoethyl fumarate, activated as pentafluoro phenol. A final saponification reaction led to the desired products (see Supporting Information).

For compound **12** the same procedure was adopted, starting from methyl 2-(4-formylphenyl)acetate.

**Scheme 1:**
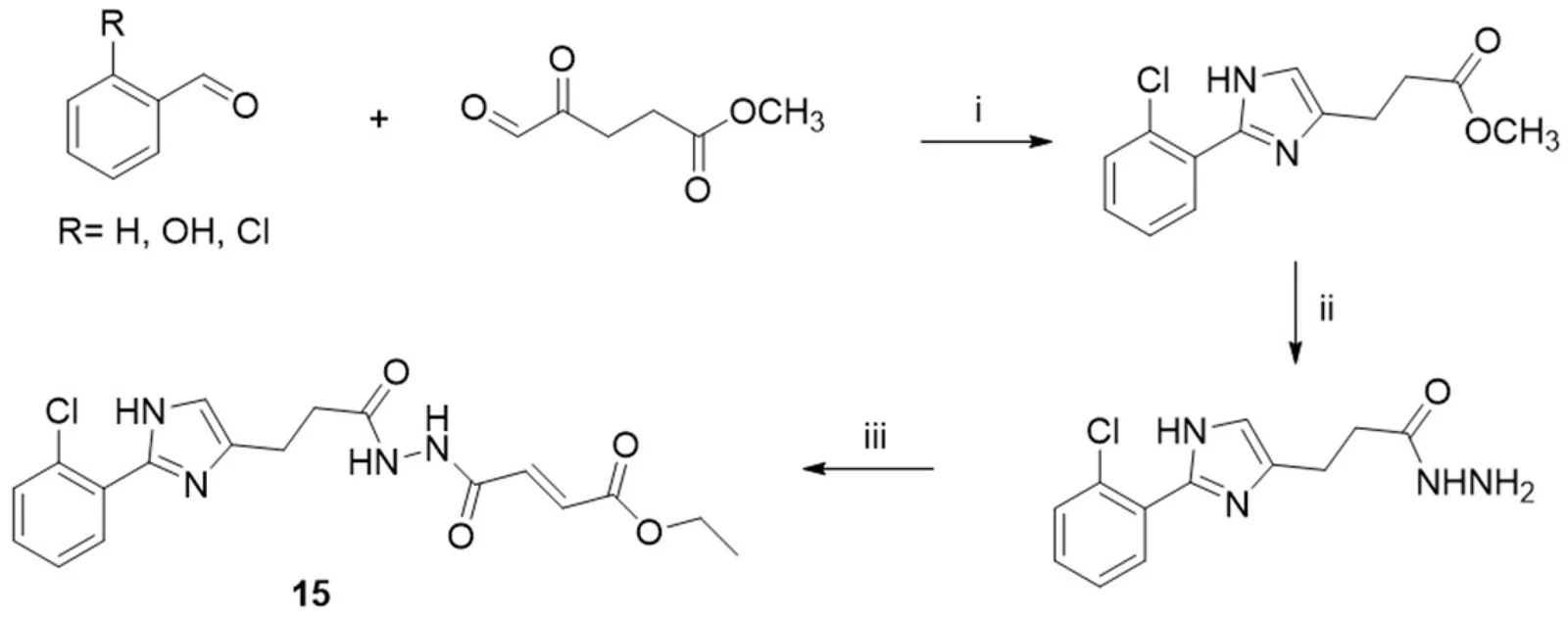
Synthesis of compound **15**. Reagents and conditions: i—NH4COOCH3; MeOH; RT; overnight; ii—NH2NH2; MeOH; mw irradiation; 20 min; iii—perfluorophenyl (E)-4-oxohept-2-enoate; EtOAc/DMSO 1:1; RT; overnight.

As a matter of fact, during the synthesis of suggested compounds **T5**–**T7** we encountered many drawbacks, in particular, in synthesizing and purifying the initial key phenyl-imidazolic intermediates. Even though we used a well-established procedure to synthesize these imidazole intermediates, as represented in Scheme 1, the yields for -H and -OH substituents were below 10% and any attempt to improve the reaction outcome was fruitless. Our best interpretation for this failure is that only 2-chloro-benzaldehyde was sufficiently activated to undergo the cyclization reaction to obtain the desired compound in an acceptable yield. With the predicted energy interaction of these target compounds being very similar, we focused our efforts on compound **T7**, with methyl 3-(2-(2-chlorophenyl)-1H-imidazol-4-yl)propanoate being the only intermediate that could be accessed with an acceptable yield and purity (Scheme 1). Our efforts led to compound **15**, which is the ethyl ester of the target compound **T7**. We faced another synthetic pitfall performing the last saponification reaction; in fact, even after varying the reaction conditions, we were not able to access the desired compound.

Since we could not obtain the carboxylic derivative (**T7**) we decided to test the ester 15 and we also decided to add compounds **13** and **14** to our set of molecules. Although preliminary docking simulations of the esterified derivatives showed reduced predicted binding affinities compared to their acidic counterparts due to the inability to form the crucial salt bridge with Arg248, we hypothesize that these esters essentially act as prodrugs. This prodrug strategy is robustly supported by recent developments in inhibitors targeting the homologous FTO enzyme [22], where the employment of ethyl ester derivatives successfully enhanced cellular penetration before undergoing intracellular hydrolysis to release the active carboxylic acid form. Based on this well-established precedent, we anticipated that these esterified compounds would be similarly hydrolyzed by ubiquitous cellular esterases in biological settings, effectively delivering the active inhibitor into the physiological compartments.

### 3.3. Inhibition of ALKBH2 Activity

An ALKBH2 activity cell-free assay was performed to investigate and compare the inhibition of active recombinant ALKBH2 protein by MV1035 and compounds **1**–**15**. ALKBH2 was incubated with methylated single-stranded DNA (ssDNA), either alone or in combination with MV1035 or compounds **1**–**15** (50 μM). After annealing with a complementary ssDNA oligonucleotide, the DNA underwent enzymatic digestion with EcoRI.

**Figure 4:**
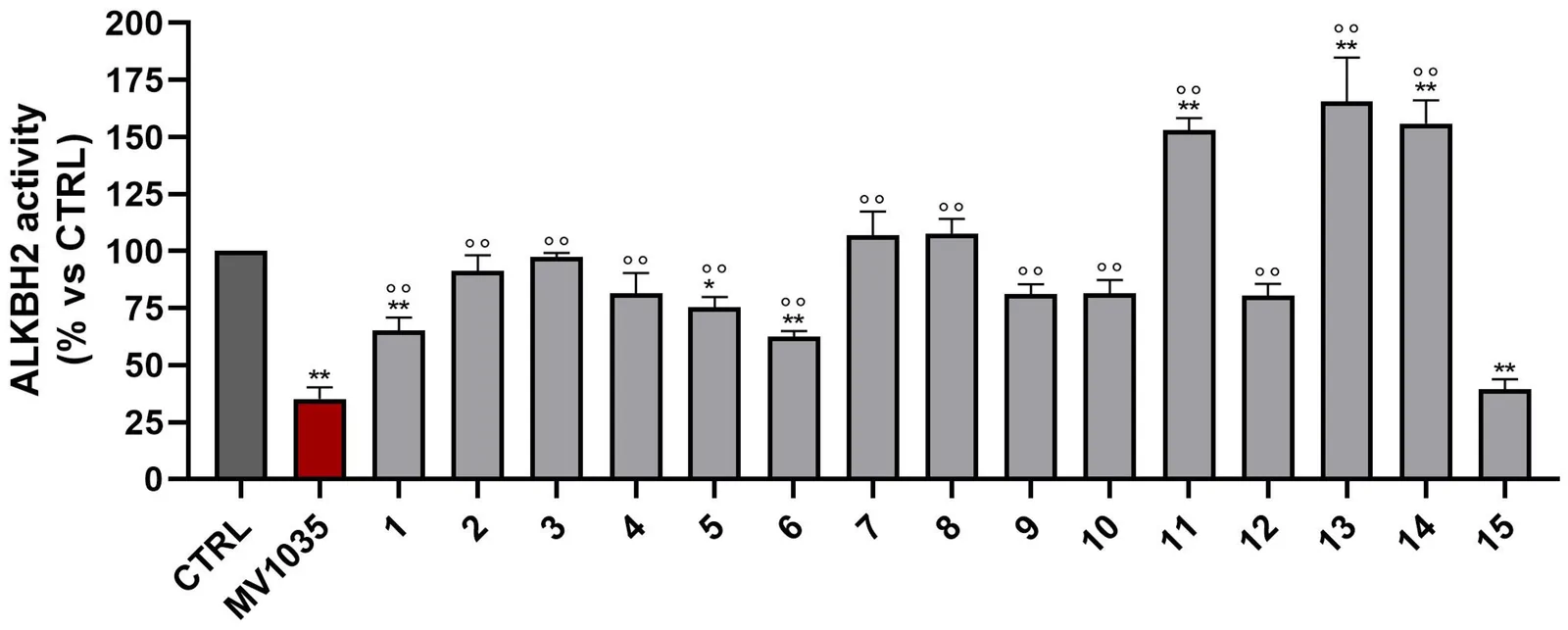
Cell-free ALKBH2 demethylase activity assay in presence of MV1035 or compounds **1**–**15** (50 μM). Data are represented as mean percentage ± SD compared to untreated controls, arbitrarily set to 100%. * *p* < 0.05 vs. CTRL; ** *p* < 0.01 vs. CTRL; °° *p* < 0.01 vs. MV1035.

Compound **15** induces a statistically significant reduction in ALKBH2 activity to a comparable extent to MV1035, inhibiting it by approximately 75%. No other compound was shown to reduce ALKBH2 activity by at least 50% and in a manner comparable to MV1035 (Figure 4).

### 3.4. Inhibition of ALKBH5 Activity

The inhibition of active recombinant ALKBH5 by MV1035 and compounds **1**–**15** (50 μM) was evaluated by dot blot analysis. ALKBH5 protein was incubated with m6A-RNA alone or with MV1035 or compounds **1**–**15** (50 μM).

**Figure 5:**
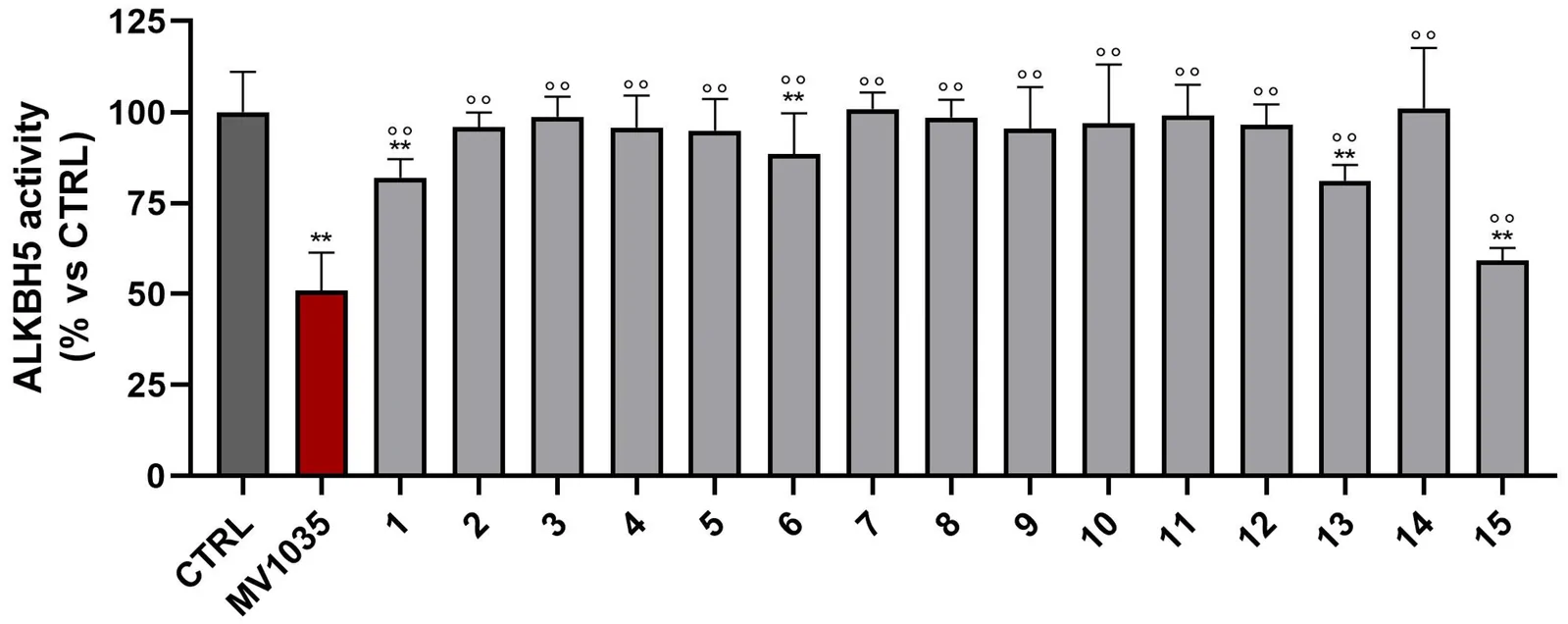
Cell-free ALKBH5 demethylase activity assay in presence of MV1035 or compounds **1**–**15** (50 μM). Data are represented as mean percentage ± SD compared to untreated controls, arbitrarily set to 100%. ** *p* < 0.01 vs. CTRL; °° *p* < 0.01 vs. MV1035.

Compounds **1**, **6**, **13** and **15** induce a statistically significant reduction in ALKBH5 activity with respect to the control. The inhibition is, however, statistically lower than that induced by MV1035; compounds **1**, **13** and **15** show a 50% effect with respect to the lead compound MV1035 (Figure 5).

### 3.5. Cytotoxic Effect on U87-MG Cells

**Figure 6:**
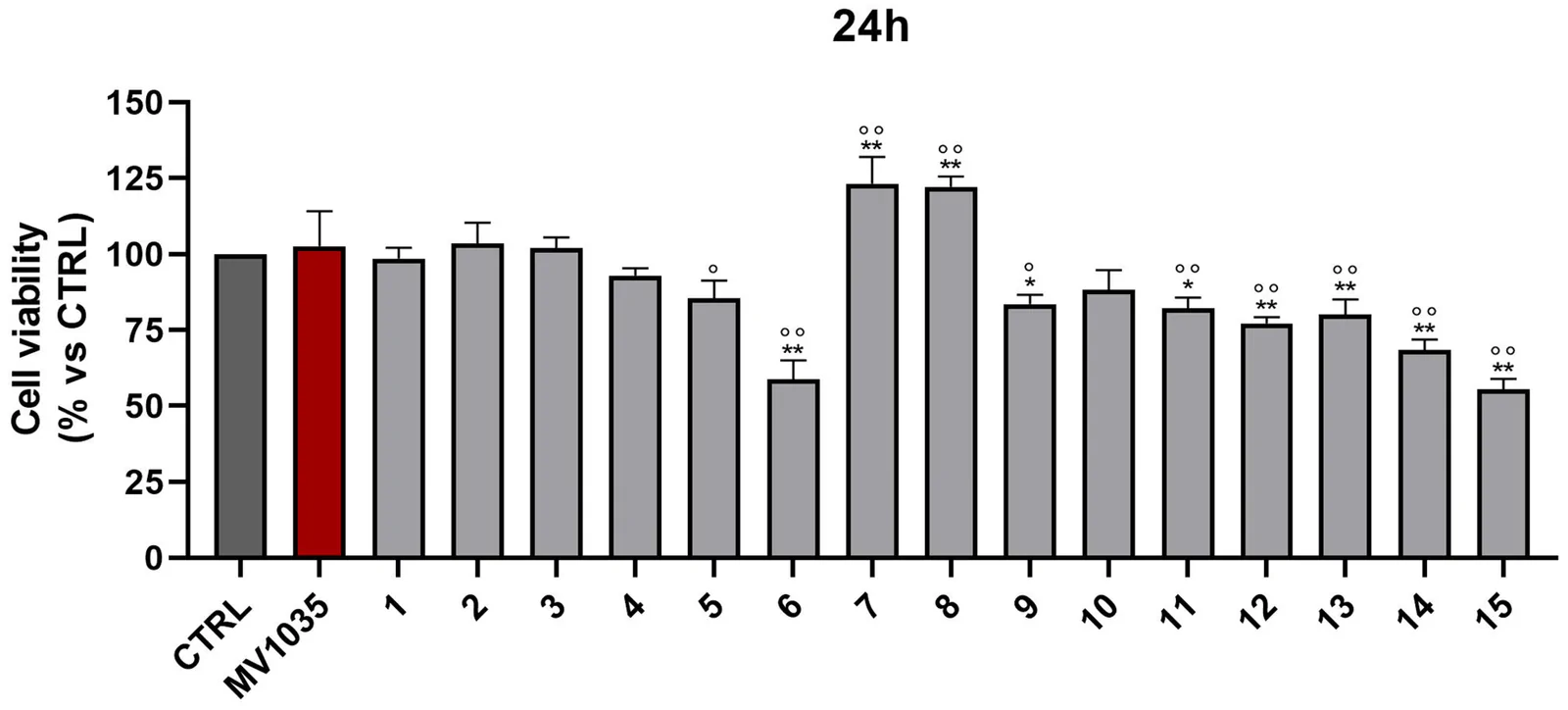
Cell viability of U87-MG cells treated with MV1035 or compounds **1**–**15** (50 μM) for 24 h. Results obtained at 48 and 72 h are represented in Supplementary Figure S1. Graph represents mean percentage ± SD of cell viability compared to untreated controls, arbitrarily set to 100%. * *p* < 0.05 vs. CTRL; ** *p* < 0.01 vs. CTRL; ° *p* < 0.05 vs. MV1035; °° *p* < 0.01 vs. MV1035.

The inhibition of U87-MG cell viability by MV1035 and compounds **1**–**15** (50 μM) was evaluated via a 3-(4,5-Dimethylthiazol-2-yl)-2,5-diphenyltetrazolium bromide (MTT) assay. After 24 h of treatment, compounds **6**, **9**, and **11**–**15** induce a statistically significant reduction in cell viability with respect to the control or to MV1035 treatment. In particular, the results indicate that compounds **6** and **15** are the most effective (Figure 6). The results after 48 and 72 h of treatment are shown in Supplementary Figure S1.

### 3.6. In Silico Prediction of Endothelial Permeability to MV1035 Analogs

To evaluate the potential of MV1035 and its derivatives to cross the blood–brain barrier (BBB), we performed an in silico evaluation of central nervous system (CNS) permeability using the Central Nervous System—Multiparameter Optimization (CNS-MPO) approach [14]. This method provides a quantitative, independent indicator of BBB penetration and is particularly suited for the rapid prioritization of compounds during early-stage drug discovery.

**Figure 7:**
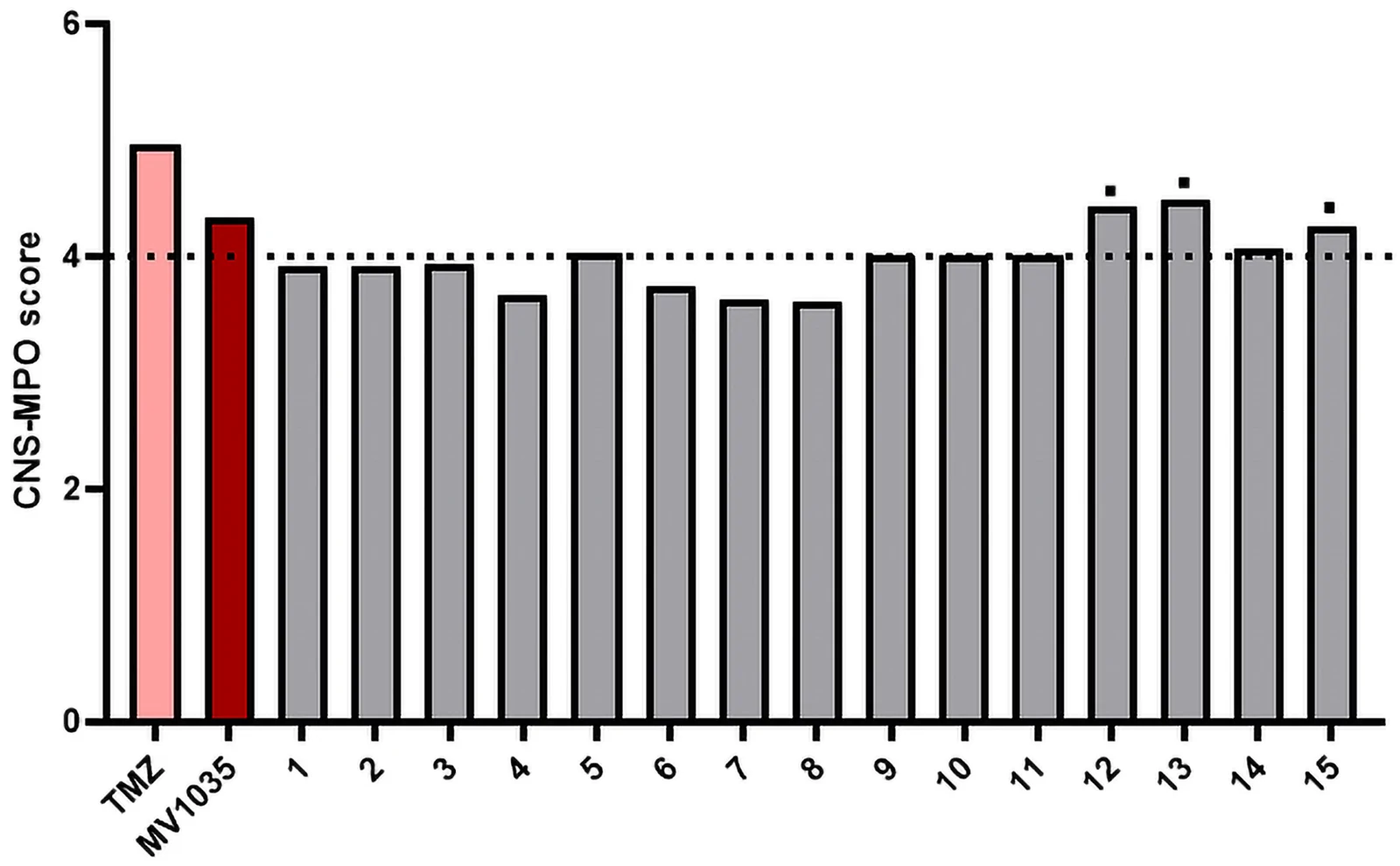
CNS-MPO score of MV1035 and its derivatives. CNS-MPO scores are shown for TMZ, MV1035, and compounds **1**–**15**. The dotted horizontal line indicates the benchmark CNS-MPO threshold of 4.0, commonly associated with favorable CNS drug-like properties. TMZ is used as a benchmark for CNS drug-like properties. Compounds marked with boxes are those that exceed the commonly accepted threshold of 4.0 (dotted line).

CNS-MPO scores were calculated for MV1035 and the newly designed derivatives and compared to that of TMZ, which was included as a clinically validated CNS-active reference compound (Figure 7). TMZ and MV1035 exhibited CNS-MPO scores higher than the threshold value of 4.0 (dotted line), serving as benchmarks for CNS drug-like properties. Compounds **1**–**15** displayed CNS-MPO values clustered around the commonly accepted threshold, indicating generally favorable physicochemical profiles for CNS exposure. Three different compounds, i.e., **12**, **13**, and **15**, exceeded this threshold and showed significantly higher CNS-MPO scores, suggesting improved balance of lipophilicity, polarity, and other key parameters relevant to CNS penetration. Overall, the majority of the series demonstrated CNS-MPO scores comparable to or exceeding the benchmark value, supporting their potential suitability for CNS-targeted drug development.

### 3.7. Identification of the Most Promising Compound for Further Investigations

**Figure 8:**
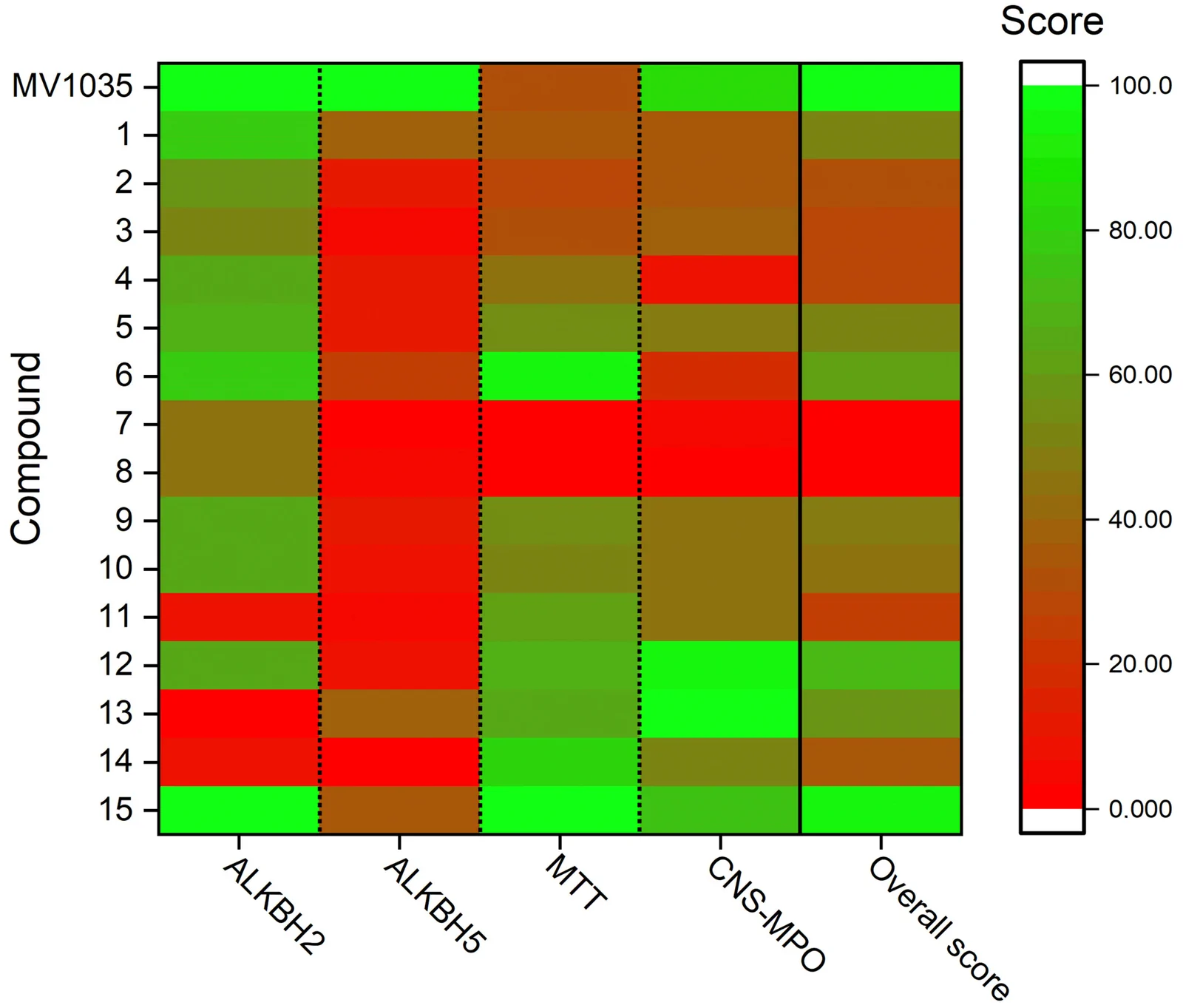
Heat map representation of the performance of MV1035 and compounds **1**–**15** across multiple screening parameters: ALKBH2 cell-free inhibition assay, ALKBH5 cell-free inhibition assay, MTT cell viability assay on U87-MG, CNS-MPO and overall score. Overall score aggregates the performance across all experiments. Scores for all metrics were normalized on a scale from 0 to 100, where 0 (red) represents the lowest measured performance/activity and 100 (bright green) represents the highest.

While TMZ has been the standard first-line treatment for glioblastoma multiforme (GBM) since 2005, its clinical utility is severely hampered by a combination of biological resistance, the blood–brain tumor barrier, and systemic toxicity [26]. In order to proceed with the study of our new compounds investigating their possible use in cotreatment with TMZ, we analyzed the data obtained in the experiments previously described to highlight the most effective overall. The heat map presented in Figure 8 clearly identifies compound **15** as the compound with the best overall score among the newly tested compounds and comparable to that of the lead compound MV1035. In particular, compound **15** turns out to be more cytotoxic against U87-MG glioblastoma cells than MV1035. Given that compound **15** emerged as the most promising candidate, we decided to assign it a distinctive and readily recognizable acronym (MV3030), rather than referring to it solely by its numerical designation, to facilitate clearer identification and citation throughout the manuscript and in the future.

### 3.8. In Vitro Blood–Brain Barrier Crossing of MV1035 and MV3030

**Figure 9:**
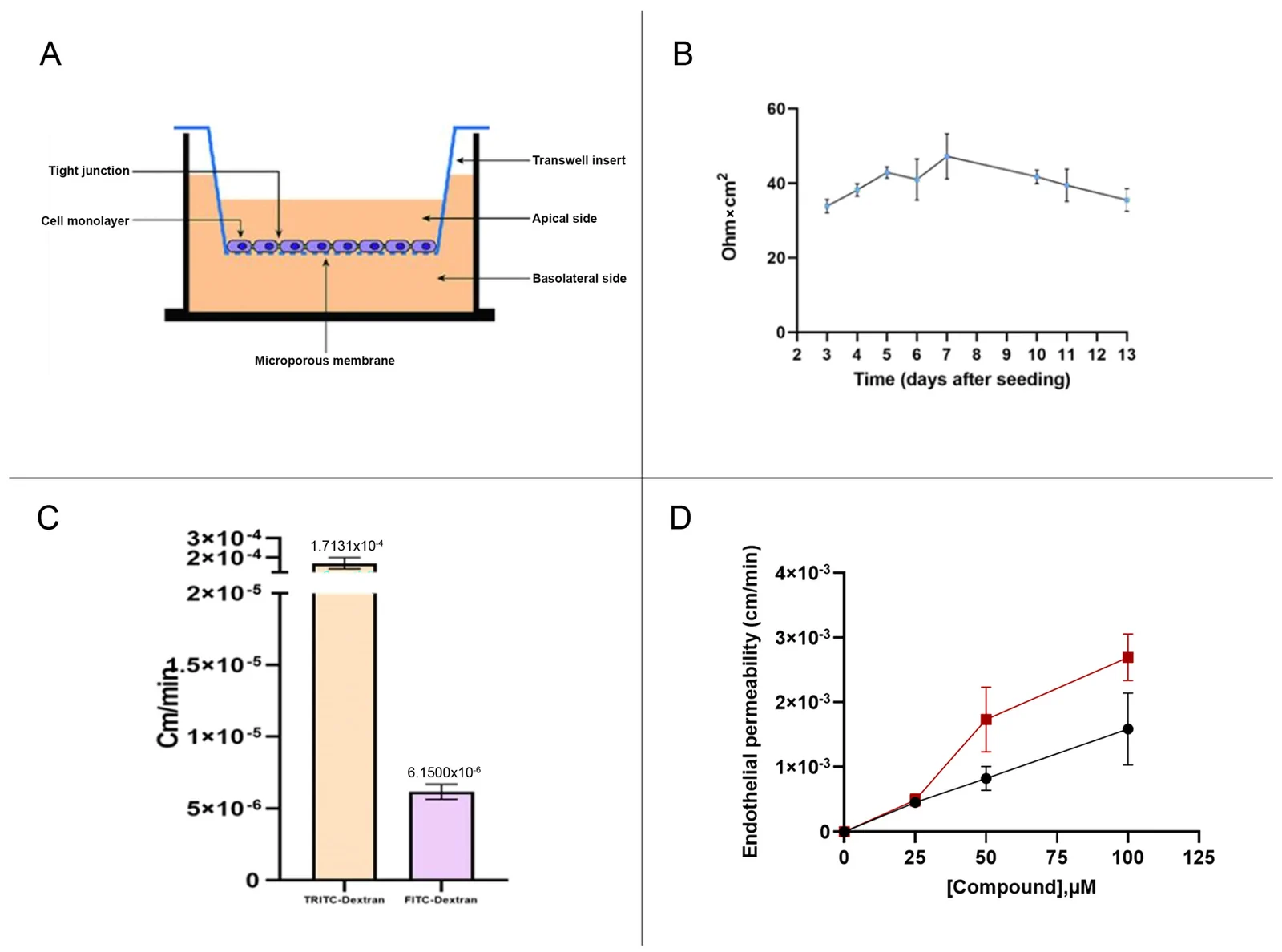
In vitro transwell model of BBB. (**A**) Graphical representation of the transwell model. (**B**) TEER values of the cell monolayers over time. (**C**) EPs to fluorescent probes. (**D**) EPs (cm/min) to MV1035 (in red) and MV3030 (in black) were assessed using a Transwell system with a confluent endothelial cell monolayer. The EP coefficient across the monolayer is reported as mean ± SD from at least three independent biological replicates. The results indicate the ability of the compounds to cross the endothelial barrier, suggesting favorable permeability properties.

To examine the BBB permeability to MV1035 and MV3030, a human endothelial cell line derived from cerebral microvessels (hCMEC/D3) was used [27,28]. As this model retains the key structural and functional properties of adult human brain endothelial cells, it is extensively used for screening the BBB transport of small molecules and nanoparticles [29,30]. When grown on transwell filters (Figure 9A), hCMEC/D3 cells exhibited transendothelial electrical resistance (TEER) values ranging from 35 Ω cm^2^ to 55 Ω cm^2^, from day 3 to day 13 after seeding, with the highest value (47 ± 6 Ω × cm^2^) occurring on day 7 after seeding (Figure 9B), according to the literature [16,28]. On day 7, the endothelial permeabilities (EPs) to TRITC-Dextran and FITC-Dextran were 1.71 ± 0.29 × 10^−4^ cm/min and 6.15 ± 0.52 × 10^−6^ cm/min, respectively (Figure 9C). These values indicate the selectivity of the cell monolayers to molecules of different MWs and correlate (R^2^ = 0.94) with the in vivo values [31].

On the day with the highest TEER value, different doses of MV1035 or MV3030 were added in the apical compartment of the transwell system and incubated at 37 °C for 3 h in PBS (pH 7.4). The amount of the compound present in the basolateral side of the transwell was determined by measuring the absorbance at 311 and 284 nm after lyophilisation and resuspension, respectively, in DMSO. A calibration curve was used for quantification. The EPs to different doses of MV1035 and MV3030 are shown in Figure 9D. The compounds exhibited measurable transendothelial transport across the cell monolayer, indicating their ability to cross the endothelial barrier. A concentration-dependent increase in EP was observed, consistent with increased transport across the in vitro BBB model at higher concentrations [32]. This concentration-dependent permeability pattern indicates that as drug concentration increases, more molecules are available for transport across the endothelial monolayer, leading to proportionally increased permeability values, a principle that is fundamental to predicting brain drug exposure and optimizing CNS-targeted therapeutic candidates [33]. However, the underlying transport mechanism cannot be inferred from these data alone, further experiments are needed. Considering that the EP of the TMZ is 5 × 10^4^ cm/min [34], these results showed that the in vitro BBB model is able to discriminate between different molecules and that the in silico tests have an excellent degree of predictability. It should be noted that the CNS-MPO score was used as a prioritization tool during compound selection and was not intended to quantitatively predict BBB permeability. The effect of MV1035 or MV3030 on hCMEC/D3 cell viability, bioelectrical and functional properties was assessed by measuring TEER and EP to fluorescent probes, after 3 h of incubation. The results show no difference in TEER and EP after incubation with the compounds compared to the control cells.

### 3.9. Cytotoxic Effect of Temozolomide, MV3030 and Temozolomide + MV3030 on U87-MG and Patient-Derived Glioma Stem Cells

To evaluate the effect of MV3030 alone and in combination with TMZ, an MTT assay was performed. We investigated the cytotoxic effect on the U87-MG glioblastoma cell line and on two different patient-derived glioma stem cells (PD-GSCs), G179 and GSC7. Although in fact, U87-MG cells represent a good cellular model for a first evaluation of anti-glioblastoma strategies, these represent biases that must be taken into account in the interpretation of the data [35]. Unlike serum-cultured lines which undergo differentiation and genetic drift, PD-GSCs preserve the stem-like properties and intrinsic drug resistance of the original malignancy, representing a superior model for validating novel therapeutics.

**Figure 10:**
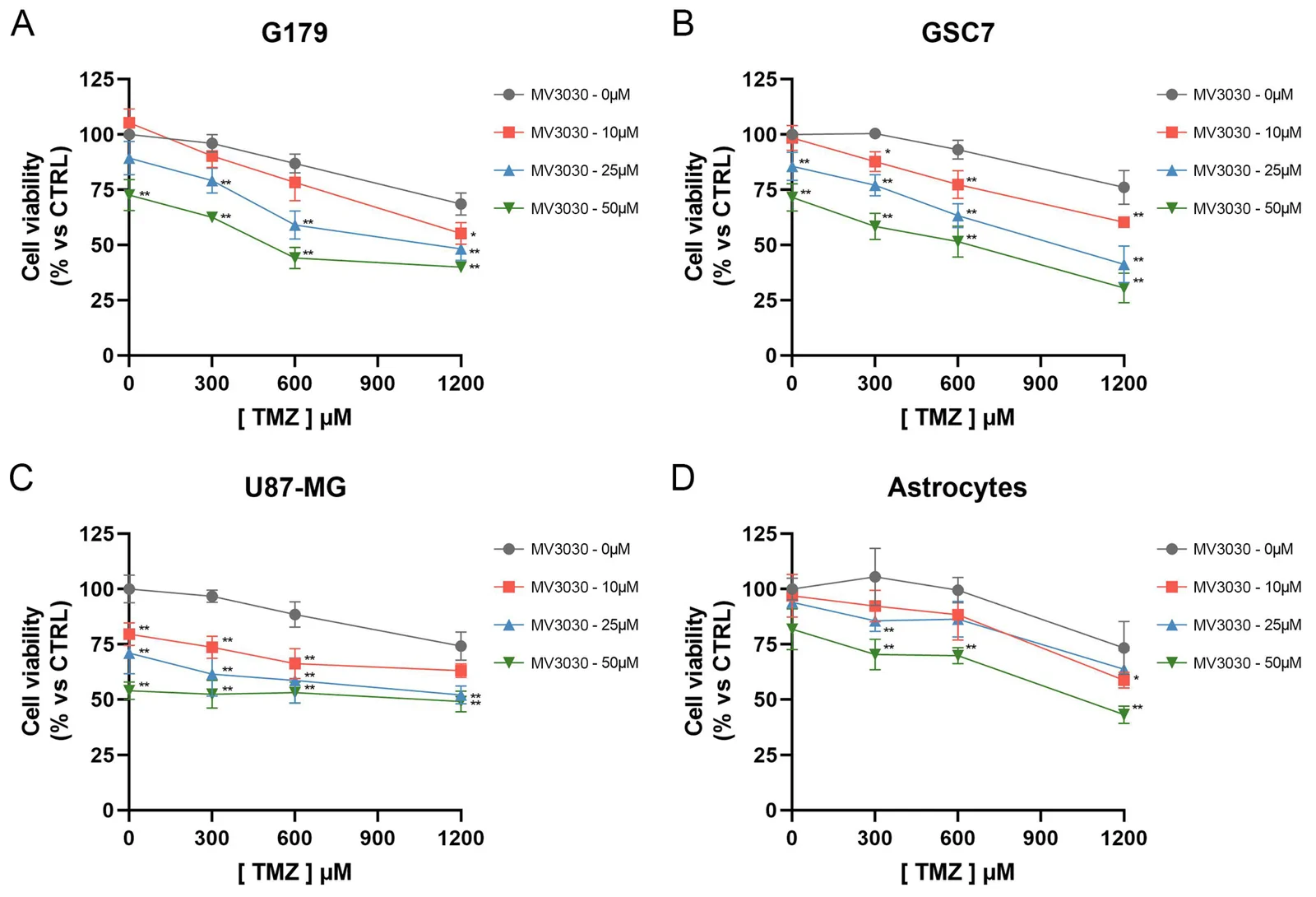
MTT assay on U87-MG (**A**), G179 (**B**), GSC7 (**C**) and astrocyte (**D**) cells treated for 24 h with MV3030 (10 μM, 25 μM, 50 μM), TMZ (300 μM, 600 μM, 900 μM, 1200 μM) and TMZ + MV3030. Data are represented as the mean percentage ± SD compared to untreated controls, arbitrarily set to 100%. * *p* < 0.05 and ** *p* < 0.01 vs. TMZ + MV3030 0 µM.

The MTT assay shows that 24 h treatment with MV3030 (50 μM) alone is cytotoxic against all tested cell lines (Figure 10); this data is noteworthy because MV1035 administered alone does not show cytotoxicity against PD-GSCs. The Results obtained from combining various concentrations of TMZ and MV3030 on PD-GSCs revealed an overall synergistic trend (G179: mean Bliss score = 14.29, mean ZIP score = 12.80; GSC7: mean Bliss score = 16.50, mean ZIP score = 15.71). Implementation of the Chou–Talalay analysis demonstrated that true synergy, characterized by a significant dose reduction (Combination Index (CI) < 1), was restricted to a specific treatment window (G179: TMZ 300 µM + MV3030 10 µM, CI = 0.81; GSC7: TMZ 300 µM + MV3030 10 µM, CI = 0.72) (Supplementary Figures S2 and S3).

In contrast, the U87-MG cell line treated with different combinations of TMZ and MV3030 exhibited an overall additive effect (mean Bliss score = 1.13, mean ZIP score = 0.87), with a synergistic window limited to the TMZ 300 µM + MV3030 10 µM combination (CI = 0.72). Furthermore, the analysis identified a strong antagonistic combination at higher concentrations (TMZ 1200 µM + MV3030 50 µM, mean Bliss score = −10.71, mean ZIP score = −10.47, CI = 2.03) (Supplementary Figure S4).

To evaluate the potential toxicity of the combined treatments, human astrocytes were included as a non-tumor healthy cell control (Figure 10D and Figure S5). Although the combination of MV3030 and TMZ induced a dose-dependent decrease in cell viability and displayed a synergistic effect in astrocytes (mean Bliss score = 12.92, mean ZIP score = 8.29), the viability curves and IC50 differed significantly from those observed in the glioblastoma models (Supplementary Figure S6). Specifically, astrocytes exhibited a markedly higher resistance to the treatment, particularly when compared to G179 and GSC7 cells, with high cell viability retained across most concentration pairs and cytotoxicity becoming apparent only at the highest dose combinations.

In Table 4, it is evident how the cotreatment with TMZ and MV3030 (25 μM and 50 μM respectively) reduces TMZ IC50. In particular the MV3030-induced TMZ IC50 decrease is greater than that induced by MV1035 in G179 PD cells [6].

### 3.10. Effect of MV3030 and MV1035 on Patient-Derived Cell Neurosphere Formation

Since one of the crucial characteristics of glioma stem cells is the capacity to form neurospheres in vitro, we evaluated this self-renewal ability after administering MV3030 or MV1035, using a sphere limiting dilution assay followed by mathematical quantification with the Extreme Limiting Dilution Analysis (ELDA) tool [21]. In G179 cells, single-agent treatments with MV1035 and TMZ significantly reduced the active stem cell fraction compared to the untreated control (CTRL) group (*p* = 5.54 × 10^−15^ and *p* = 1.42 × 10^−13^, respectively). Strikingly, treatment with MV3030 monotherapy led to a complete absence of neurosphere formation across all tested cell doses (300, 600, and 1200 cells/well), with no sphere-forming cells detected under these experimental conditions (*p* = 9.23 × 10^−18^ vs. CTRL; upper-bound frequency estimate derived from ELDA 95% confidence intervals). Similar complete suppression was achieved by the combined treatment of TMZ with either MV1035 or MV3030 (*p* = 9.23 × 10^−18^ vs. CTRL). Due to the lack of sphere growth in these highly effective groups, pairwise comparisons showed no statistically significant differences between MV3030 alone and either MV1035 monotherapy (*p* = 0.238) or the combined regimens (*p* = 1.00).

**Figure 11:**
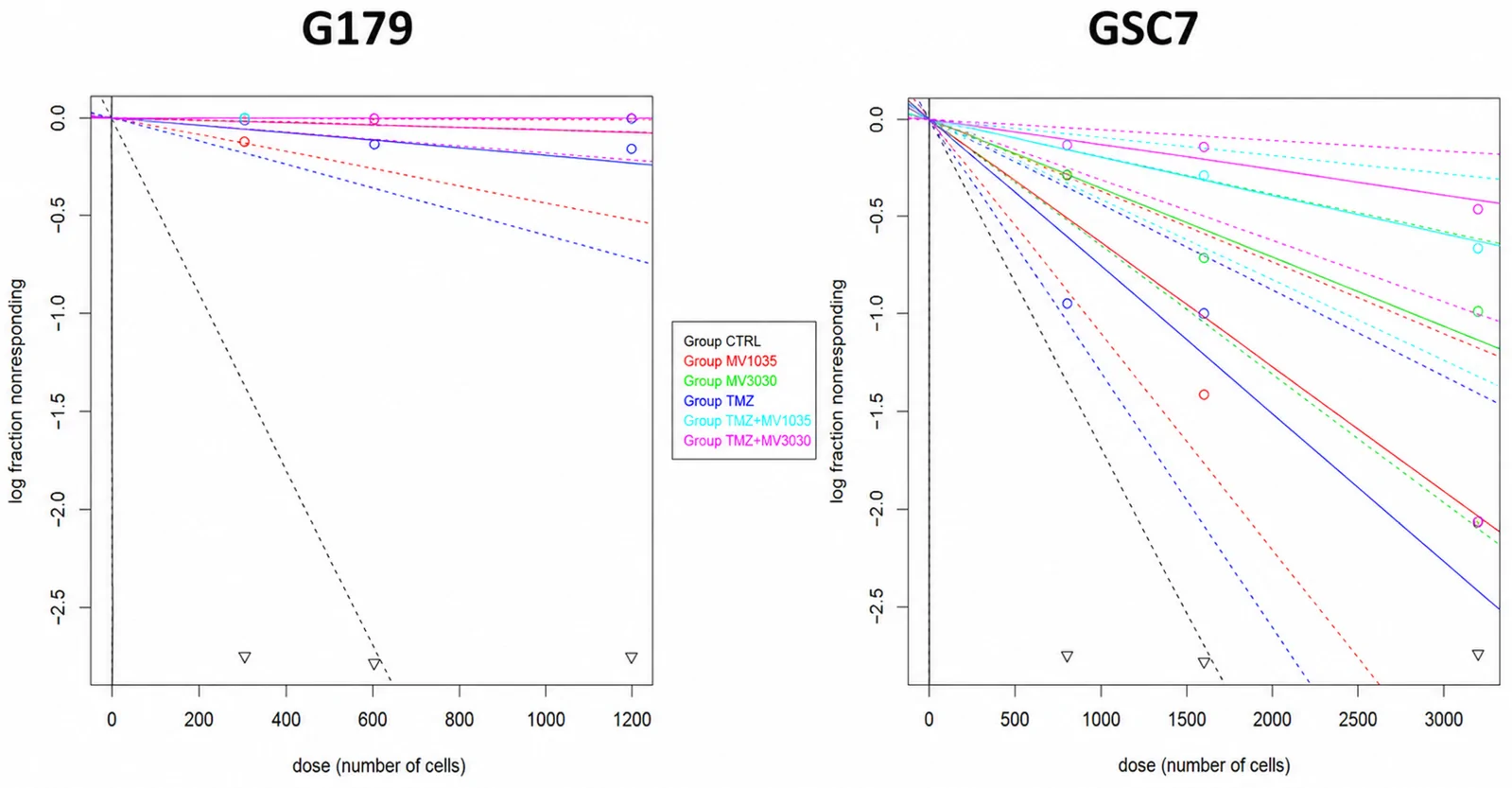
Evaluation of stem cell frequency via limiting dilution assay (LDA). Data analysis was performed using the software-guided Extreme Limiting Dilution Analysis (ELDA) tool [21]. The number of initially seeded cells per well (dose, *X*-axis) is plotted against the log fraction of non-responding wells (wells without detected spheres, *Y*-axis). The slope of each linear regression represents the log-active stem cell fraction (clonogenic frequency) for each respective treatment group. Dotted lines represent the 95% confidence intervals. For groups lacking neurosphere formation, upper-bound frequencies and lower 95% confidence limits were calculated using the Poisson model. Circles represent the observed log fraction of non-responding wells for each cell dose, indicating the actual experimental data points mapped against the mathematical trendline. Solid lines closer to the horizontal axis (lower slope) indicate a reduction in the frequency of sphere-forming cells under specific pharmacological treatments compared to the control (CTRL) group. Circles represent the observed log fraction of non-responding wells for each cell dose, indicating the actual experimental data points mapped against the mathematical trendline. Open triangles on the baseline represent doses where all replicates responded (100% sphere formation).

The inhibitory effect of the compounds was less absolute in the more resistant GSC7 line, allowing for finer discrimination of cooperative effects. In these cells, single-agent administrations of TMZ, MV1035, and MV3030 significantly decreased stem cell frequencies compared to the CTRL group (*p* = 0.000205, *p* = 5.52 × 10^−5^, and *p* = 1.8 × 10^−7^, respectively), with MV1035 and MV3030 showing statistically equivalent potencies (*p* = 0.162). Crucially, the addition of TMZ to the ALKBH inhibitors resulted in a cooperative shift in sphere-forming suppression. Combined treatment with TMZ + MV1035 significantly reduced the active stem cell fraction compared to MV1035 alone (1/5091 cells vs. 1/1571 cells; *p* = 0.00997). In line with the additive profile observed in cytotoxicity assays, this trend was even more pronounced with the TMZ + MV3030 combination, which induced a marked drop in clonogenic frequency compared to MV3030 monotherapy (1/7687 cells vs. 1/2816 cells; *p* = 0.0551, showing a strong trend toward significance), outperforming the other therapeutic arms and highlighting increased sensitivity of GSC7 cells to temozolomide upon ALKBH inhibition (Figure 11 and Figure S7).

### 3.11. Possible Perturbation of FoxM1 and Wnt/β-Catenin Pathways

Zhang et al. (2017) have demonstrated that ALKBH5 activity maintains tumorigenicity of GBM stem-like cells by sustaining the expression of the transcription factor FoxM1 [36]. Considering the efficacy of MV1035 and MV3030 on U87-MG cells and on PD cells and their ability to inhibit ALKBH5 activity, we investigated their possible modulation of FoxM1 protein expression and the consequent perturbation of the Wnt/β-Catenin pathway. In particular, by immunoblotting, we investigated FoxM1, β-Catenin, Survivin, and Cyclin-E protein expression.

**Figure 12:**
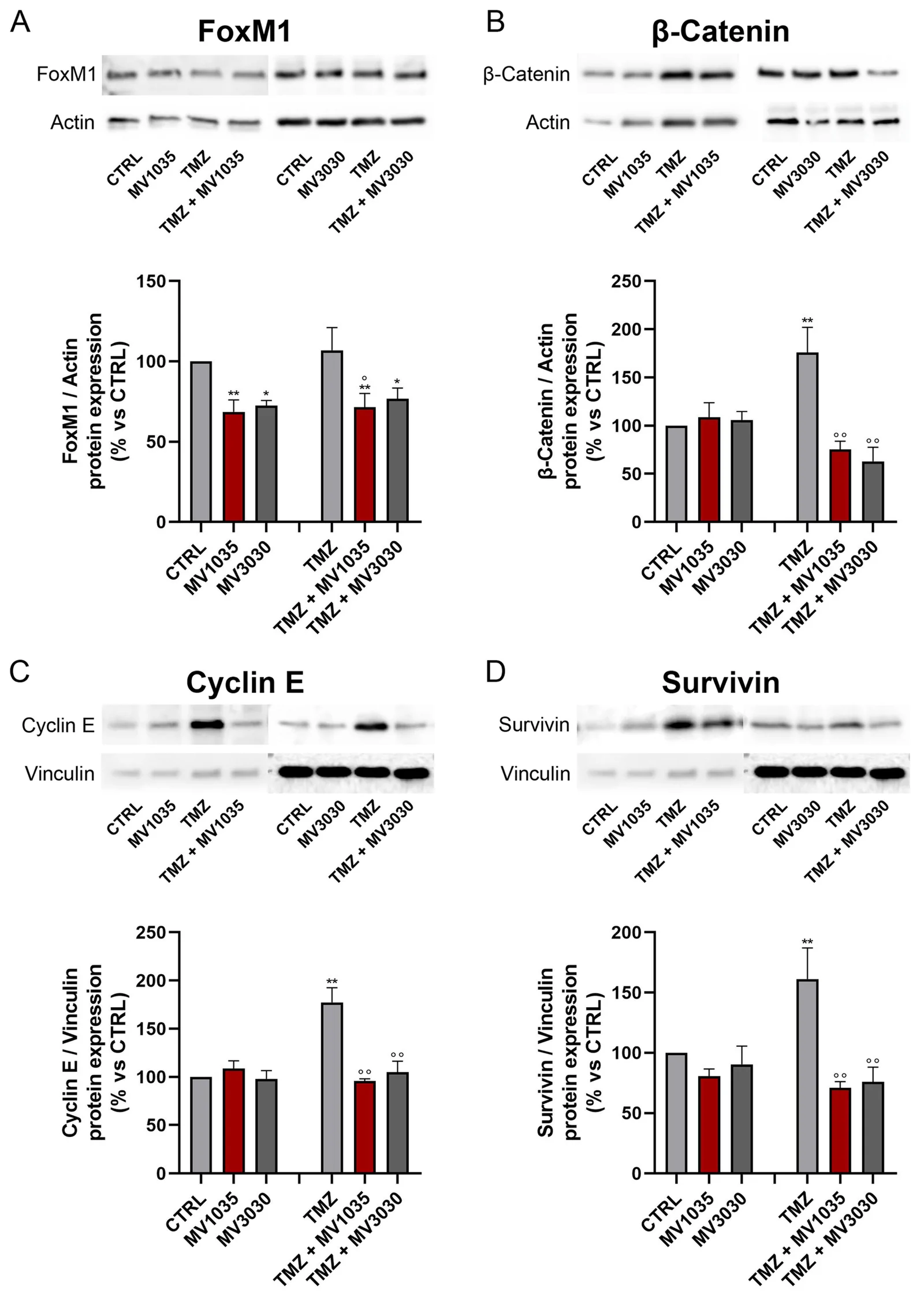
Representative Western blot images and quantification of FoxM1 (**A**), β-Catenin (**B**), Cyclin E (**C**) and Survivin (**D**). G179 PD-GBM cells were treated with MV1035 (50 µM), MV3030 (50 μM), TMZ (600 µM) and their combinations for 24 h. Graphs represent mean percentage ± SD compared to untreated controls, arbitrarily set to 100%. * *p* < 0.05 vs. CTRL; ** *p* < 0.01 vs. CTRL; ° *p* < 0.05 vs. TMZ; °° *p* < 0.01 vs. TMZ. Blots in (**C**,**D**) were similarly processed using dedicated membranes per group, and were cut and probed for Cyclin E, Survivin, and Vinculin. All antibody specificities were previously validated prior to membrane cutting.

**Figure 13:**
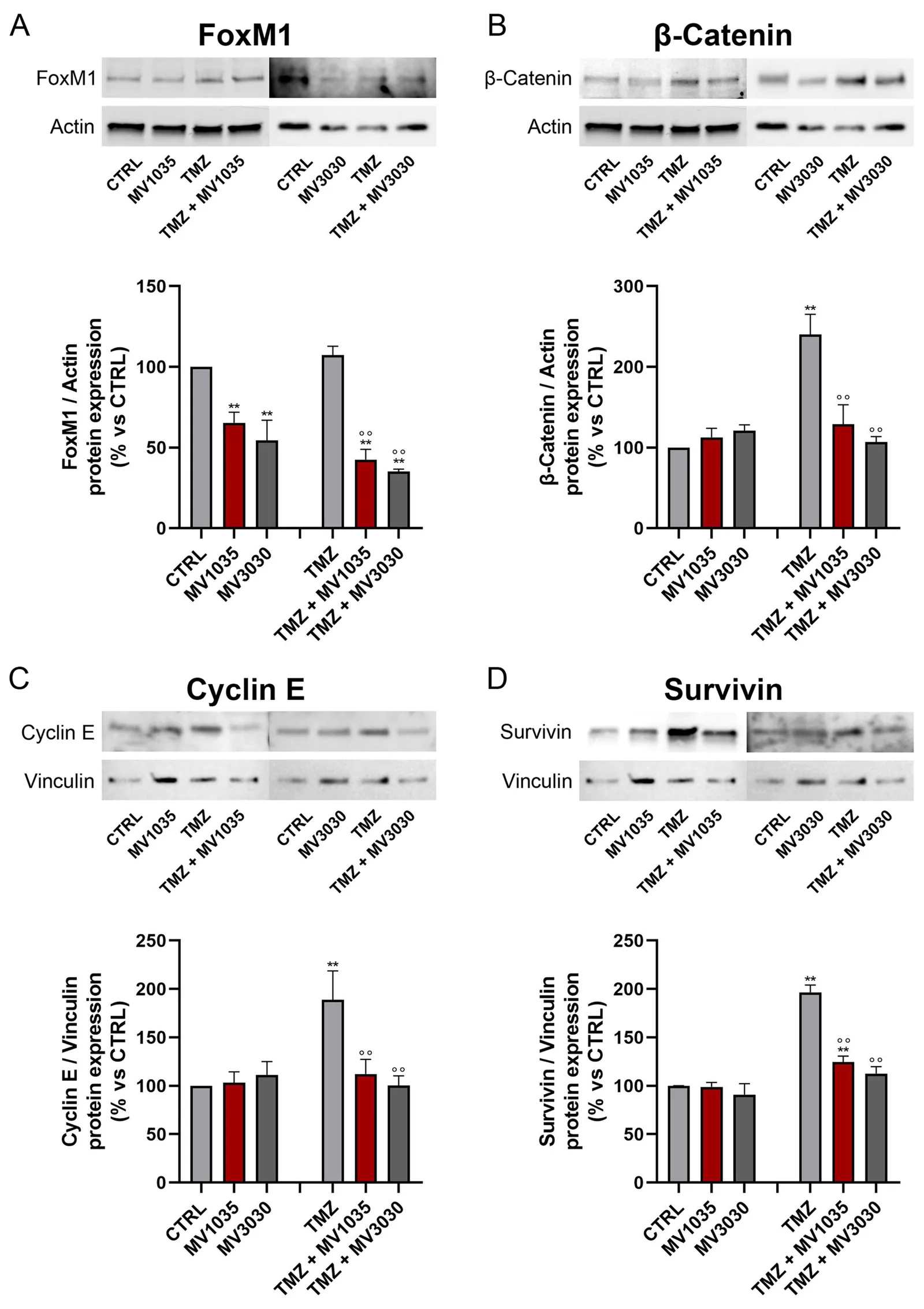
Representative Western blot images and quantification of FoxM1 (**A**), β-Catenin (**B**), Cyclin E (**C**) and Survivin (**D**). GSC7 PD-GBM cells were treated with MV1035 (50 µM), MV3030 (50 μM), TMZ (600 µM) and their combinations for 24 h. Graphs represent mean percentage ± SD compared to untreated controls, arbitrarily set to 100%. ** *p* < 0.01 vs. CTRL; °° *p* < 0.01 vs. TMZ. Blots in (**A**,**B**) were processed using one membrane per experimental group (MV1035 and MV3030), and were cut and probed for FoxM1, β-Catenin, and Actin. Blots in (**C**,**D**) were similarly processed using dedicated membranes per group, and were cut and probed for Cyclin E, Survivin, and Vinculin. All antibody specificities were previously validated prior to membrane cutting.

G179 and GSC7 PD cells were treated for 24 h with TMZ (600 μM) or with MV3030 or with MV1035 (50 μM) or their combinations. Immunoblotting demonstrates that both MV1035 and MV3030 reduce, in a statistically significant way, the expression of FoxM1 protein with respect to control cells, and the cotreatments decrease, in a statistically significant way, the expression of FoxM1 protein with respect to TMZ-treated cells (Figure 12A and Figure 13A).

Moreover, both MV1035 and MV3030 counteract, in a statistically significant way, the increase in TMZ-induced β-Catenin, Survivin, and Cyclin-E protein expression in both PD cells (Figure 12 and Figure 13).

**Figure 14:**
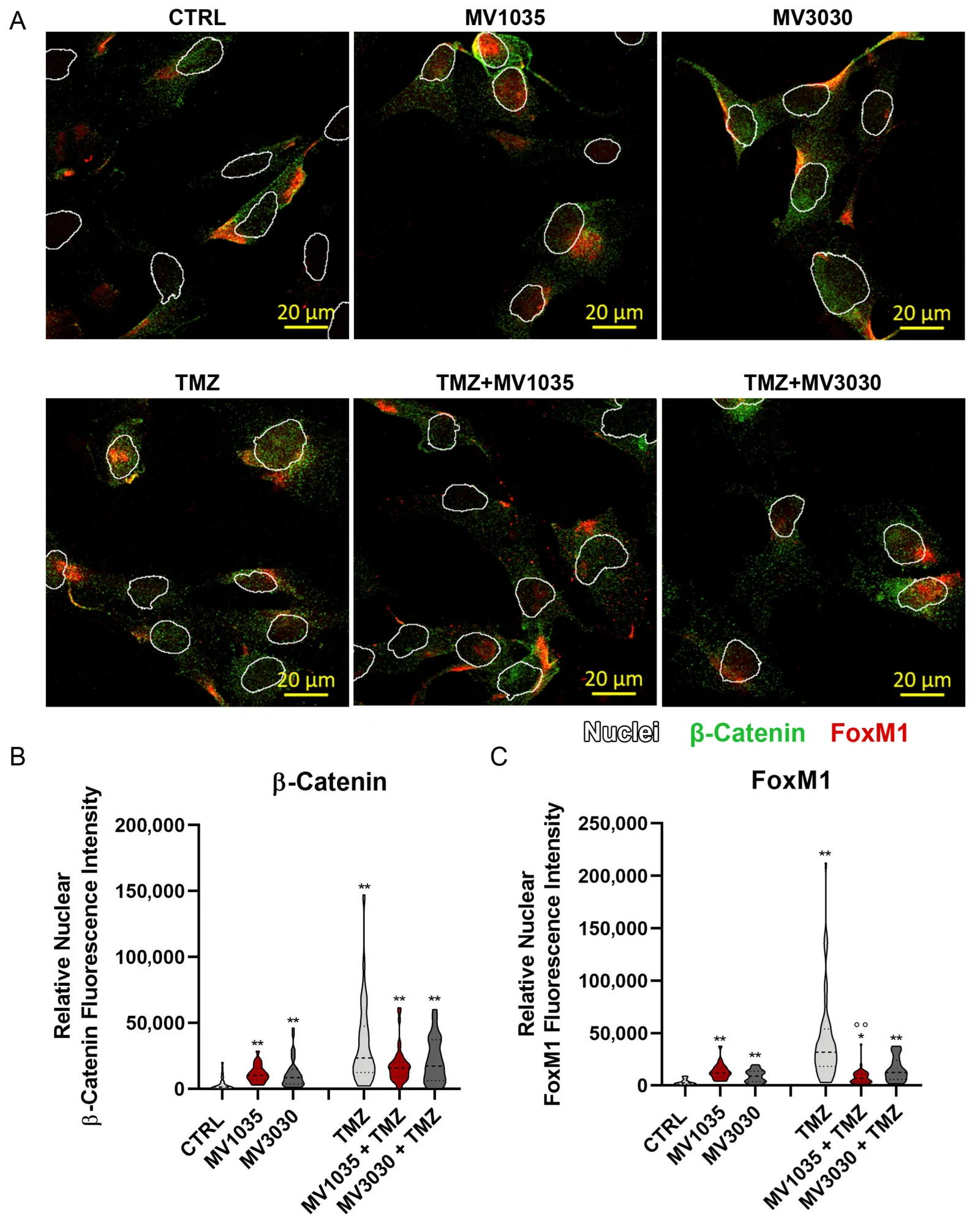
Representative immunofluorescence images of FoxM1 and β-Catenin in G179 cells (**A**). G179 PD GBM cells were treated with MV1035 (50 µM), MV3030 (50 µM), TMZ (600 µM), and their combinations for 24 h. Samples were immunostained for FoxM1 (red) and β-catenin (green). Nuclei were counterstained with DAPI (to better visualize the localization of FoxM1 and β-catenin, only the nuclear perimeter is outlined in white). Violin plots represent the distribution of relative nuclear fluorescence intensity of β-catenin (**B**) and FoxM1 (**C**). The dashed line indicates the mean, while the dotted lines represent the upper and lower quartiles. * *p* < 0.05 vs. CTRL; ** *p* < 0.01 vs. CTRL; °° *p* < 0.01 vs. TMZ.

**Figure 15:**
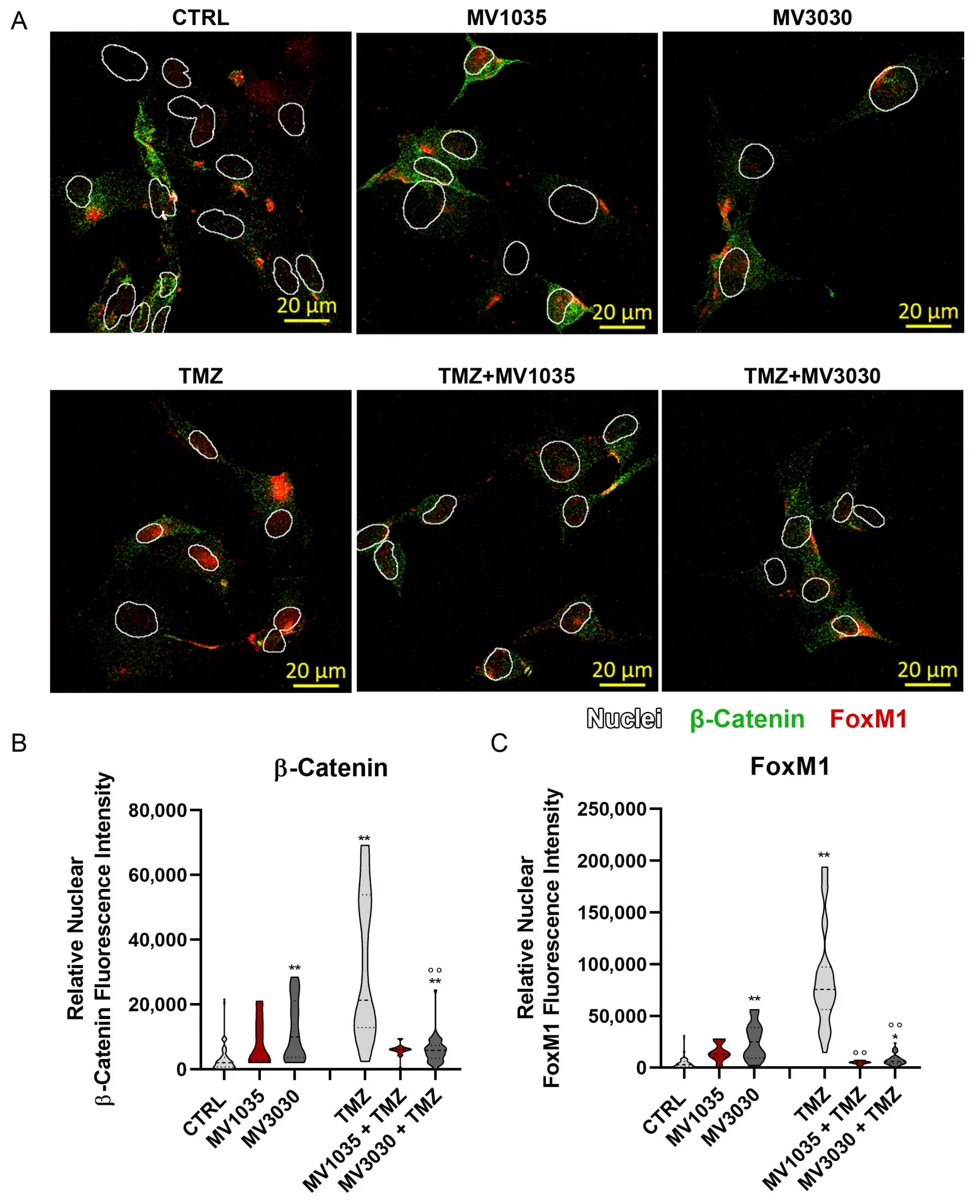
Representative immunofluorescence images of FoxM1 and β-Catenin in GSC7 cells (**A**). GSC7 PD GBM cells were treated with MV1035 (50 µM), MV3030 (50 µM), TMZ (600 µM), and their combinations for 24 h. Samples were immunostained for FoxM1 (red) and β-catenin (green). Nuclei were counterstained with DAPI (to better visualize the localization of FoxM1 and β-catenin, only the nuclear perimeter is outlined in white). Violin plots represent the distribution of relative nuclear fluorescence intensity of β-catenin (**B**) and FoxM1 (**C**). The dashed line indicates the mean, while the dotted lines represent the upper and lower quartiles. * *p* < 0.05 vs. CTRL; ** *p* < 0.01 vs. CTRL; °° *p* < 0.01 vs. TMZ.

In agreement with these data, anti β-Catenin immunohistochemistry demonstrates that MV3030 and MV1035 -induced reduction in FoxM1 expression correlates with an increase in β-Catenin cytosolic localization (Figure 14 and Figure 15).

Comparable results were obtained in U87-MG cells across both Western blot (Supplementary Figure S8) and immunofluorescence assays (Supplementary Figure S9).

## 4. Discussion

In the context of developing novel therapeutic strategies against glioblastoma, the identification and characterization of new inhibitors targeting ALKBH5 and ALKBH2 are gaining increasing attention [4,37,38,39,40,41,42]. ALKBH2 functions as a DNA repair enzyme that removes alkylation damage, including N3-methylcytosine and N1-methyladenine, thereby contributing to resistance to alkylating agents [43,44]. In particular, its role in mediating resistance to TMZ has been demonstrated through ALKBH2-targeted siRNA approaches [41]. Moreover, ALKBH2 expression is significantly upregulated in glioblastoma cell lines and PD glioblastoma samples compared with normal tissues [39,42], suggesting a role not only in drug resistance but also in epigenetic regulation.

On the other hand, ALKBH5 is frequently overexpressed in glioblastoma and plays a critical role in maintaining glioma stem cell self-renewal by demethylating transcripts such as FoxM1 and WNT-related genes [45].

Our computational studies guided the rational design of a new class of putative ALKBH2 inhibitors based on the MV3006 scaffold. The first-generation compounds primarily targeted the DNA base-binding region and exhibited moderate affinity. Incorporation of a fumarate hydrazide moiety, inspired by known FTO inhibitors, enabled the design of a second-generation series, **T1**–**T7**, with significantly improved predicted binding affinities. These improvements are attributed to effective chelation of the Mn(II) ion and establishment of key interactions within the cofactor-binding pocket, as supported by docking results. However, as previously discussed, the compounds synthesized and tested, as reported in Table 2, partially differ from the initially designed structures due to limitations related to synthetic accessibility.

Among these synthesized derivatives, MV3030 emerged as our most promising candidate. Interestingly, enzymatic assays revealed that while 50 μM MV3030 maintains robust inhibition against ALKBH2, it exhibits reduced inhibitory activity towards ALKBH5 compared to the parent compound 50 μM MV1035.

In more detail, the evaluation of ALKBH2 and ALKBH5 enzymatic activity revealed dose-dependent inhibition for both compounds (Supplementary Figure S10A,B). However, assessments of ALKBH2 and ALKBH5 activity demonstrated that MV1035 was more effective at inhibiting enzyme activity than MV3030 (ALKBH2 IC50 value of 25.65 ± 4.33 µM versus 45.60 ± 5.78 µM, *p* = 0.0001; ALKBH5 IC50 of 31.24 ± 7.33 µM versus 66.25 ± 14.50 µM, *p* = 0.0001) (Supplementary Figure S11).

Although molecular docking initially predicted similar binding affinities for the second-generation scaffolds against both isoforms, we retrospectively analyzed the predicted binding modes to rationalize the target selectivity observed in vitro. Visual inspection of the DNA-binding cavities reveals a critical structural difference between the two enzymes (Supplementary Figure S11). In ALKBH2, residue Phe124 provides optimal stabilization to the aromatic core of the ligands via π-π stacking. In ALKBH5, the corresponding residue is Tyr141, whose aromatic ring is slightly shifted due to a specific hydrogen bond network engaging its hydroxyl group with neighboring residues (Arg130 and Asp125).

The parent compound MV1035, being smaller and lacking extended structural constraints, possesses sufficient conformational freedom to adapt to this shifted aromatic plane. In contrast, the active form of MV3030 is tightly anchored to the bottom of the cofactor cavity by the fumarate hydrazide chain. This strong distal constraint restricts the flexibility of the phenyl-imidazole scaffold, preventing it from properly adjusting to the Tyr141 plane. Consequently, the stabilizing π-π stacking interaction is impaired in ALKBH5. This structure-based rationale explains why the fumarate hydrazide anchoring, while boosting ALKBH2 target engagement, induces a conformational penalty in ALKBH5, ultimately leading to the observed differences in enzymatic inhibition.

The computational study and synthesis of novel ALKBH2 and ALKBH5 inhibitors performed in our study allowed us to identify MV3030 as a suitable starting point for further development of novel inhibitors. The synthesized compound MV3030 functions as an ester prodrug of the in silico designed carboxylic acids (T-series). This prodrug approach was rationally selected to mask the polar carboxylic moiety, a strategy previously validated in the development of structurally related FTO inhibitors [22], where ethyl ester derivatives successfully improved cellular uptake before undergoing intracellular hydrolysis to the active acid form. Consistent with this rationale, MV3030 demonstrated the ability to cross the endothelial barrier in vitro, supporting its potential for brain-targeted applications requiring BBB permeability.

Surprisingly but very interestingly, despite the above-reported data regarding the inhibition of ALKBH2 and ALBKH5, MV3030 displayed intrinsic cytotoxic activity in U87-MG cells as well as in G179 and GSC7 PD cells, even in the absence of TMZ, an effect not observed with MV1035. Consistent with this, mathematical modeling of our limiting dilution assays via ELDA software demonstrated that both ALBH inhibitors drastically impair the self-renewal and sphere-forming capacity of patient-derived cells, showing massive significance against the untreated controls (*p* < 0.001). Direct pairwise comparisons showed that MV3030 and MV1035 monotherapies were statistically equivalent in inhibiting sphere formation (*p* = 0.397 for G179; *p* = 0.162 for GSC7). However, their cooperative impact was line-dependent. In G179 cells, MV3030 monotherapy was sufficient to completely eliminate detectable sphere-forming capacity under the tested conditions, matching the maximum effect observed with the combined regimens. Conversely, in the more resistant GSC7 line, the combination of TMZ and MV3030 increased efficacy compared to MV3030 alone (*p* = 0.0551 for trend; *p* = 0.00116 vs. TMZ monotherapy), outperforming the TMZ + MV1035 combination.

These findings, which highlight a powerful reduction in clonogenic sphere-forming frequency despite minor MV3030-induced inhibition of ALKBH5, could be attributed to higher intracellular concentrations of the compound due to enhanced influx or reduced efflux. Alternatively, it cannot be excluded that this agent may also act on an unidentified off-target. In this context, among all the possible off-targets, it would be interesting to investigate whether MV3030 can inhibit JmjC domain-containing histone demethylases, proteins involved in the epigenetic regulation of chromatin that share a catalytic mechanism based on the Fe(II)/2-OG cofactor. Some of these proteins are overexpressed in glioblastoma and their knockdown could reduce glioblastoma malignancy [46]. This noteworthy aspect warrants further investigation to fully elucidate the MV3030 effect.

On the other hand, molecular characterization of the effect of MV3030, alone, or in combination with TMZ, highlighted the MV3030-induced reduction in the expression of FoxM1 and the modulation of the molecular pathway Wnt/β-catenin. In agreement with Zhang et al. (2017), who demonstrated that ALKBH5 preserves the tumorigenicity of GBM stem-like cells by sustaining the expression of the transcription factor FoxM1, our data suggest that the MV3030-induced reduction in FoxM1 expression may be due to the inhibition of ALKBH5 [36]. Furthermore, Zhang et al. (2011) and Gong and Huang (2012) have demonstrated that FoxM1, binding to β-Catenin and thus promoting β-Catenin translocation from cytoplasm into the nucleus, plays a role in Wnt/β-Catenin signaling [47,48]. The Wnt/β-catenin signaling pathway is strongly implicated in glioblastoma progression where it regulates stemness, invasion, and drug resistance [49,50]. In several models it has been shown that the Wnt/β-catenin signaling pathway is amplified by TMZ and its inhibition improves TMZ effectiveness [51,52]. Similarly, Dai et al. (2010) have demonstrated that expression of FoxM1B in human glioma tissues is directly correlated with glioma grade and with the cell tumorigenicity and invasiveness [53].

Notably, we demonstrated that MV3030 was able to reduce: (a) the expression of FoxM1; (b) the TMZ-induced nuclear translocation of β-catenin; and (c) the expression of Survivin and cyclin E, suggesting a potential mechanism by which MV3030 is able to enhance TMZ’s effect in both PD cell lines.

Survivin and Cyclin E represent two proteins whose expression is regulated by the FoxM1-WnT/β-Catenin axis pathway. Survivin, an anti-apoptotic protein, is frequently upregulated in response to TMZ treatment and radiation, inducing the phosphorylation of Survivin, and enhances radioresistance in glioblastoma cells [54,55,56]. Moreover, Survivin expression is a negative prognostic factor [56]. The expression of Cyclin E, a regulatory protein of the progression of G0-S, is increased in several cancers [57]. In particular, in NHA-E6/E7/hTERT, glioma cells, overexpressing FoxM1B, have demonstrated that the expression of Cyclin E is induced through Akt activation [53]. On the other hand inhibition of Cyclin E1 is shown to be effective in increasing sensitivity to TMZ [58,59].

## 5. Conclusions

Taking all our data together, MV3030 should be considered a promising multi-targeted molecule to enhance GSC sensitivity to TMZ and suppress functional self-renewal capacity in patient-derived models. Although this study evaluated stemness suppression strictly through functional limiting dilution assays (ELDAs), future developments will aim to optimize target affinity while validating the molecular markers (e.g., CD133, SOX2, OLIG2) associated with this phenotypic blockade.

Moreover, in order to further characterize the effect of MV3030, future experiments should identify possible off-target and molecular mechanisms (m6A-dependent mRNA stability or translational/post-translational modification) by which the FoxM1-WnT/β-Catenin pathway is perturbed by MV3030.

In any case, further development of this molecule will be aimed at improving its affinity and selectivity for the desired targets.

## Figures and Tables

**Table 1:** Chemical structures of the ALKBH2 inhibitors evaluated computationally in this study. Compounds MV1035, MV3003-MV3012 and **1**–**8** constitute the initial set investigated.

Compound	Structure	Compound	Structure
MV1035	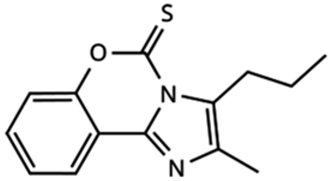	MV3012	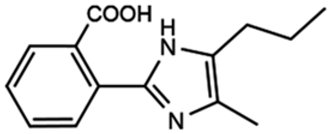
MV3003	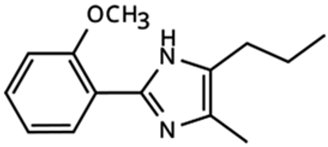	**1**	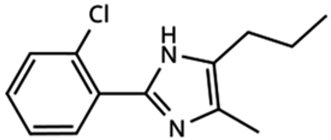
MV3004	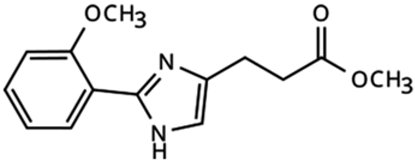	**2**	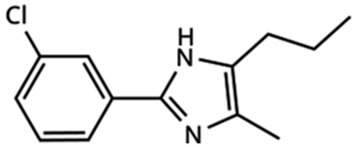
MV3005	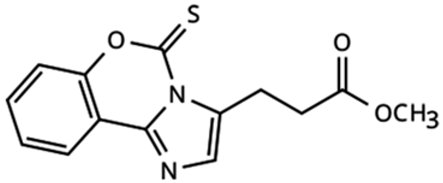	**3**	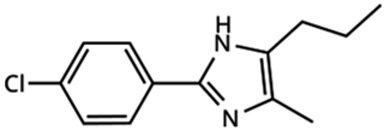
MV3006	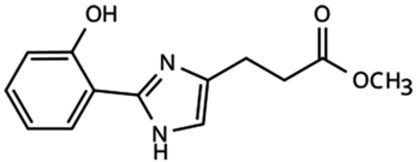	**4**	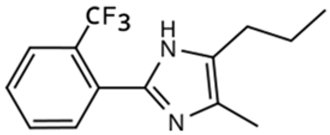
MV3007	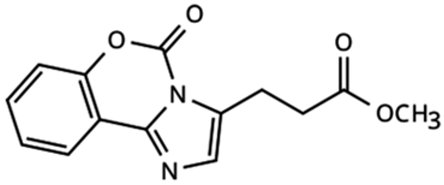	**5**	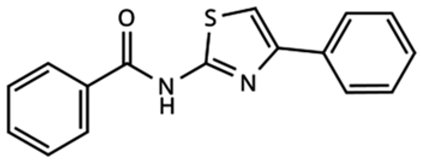
MV3009	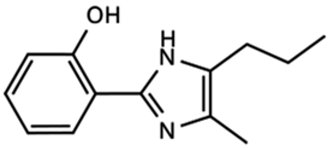	**6**	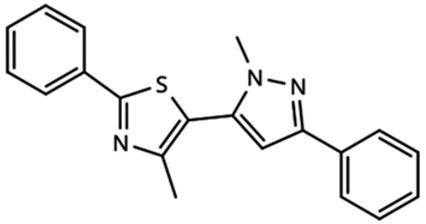
MV3010	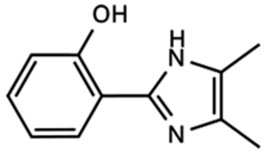	**7**	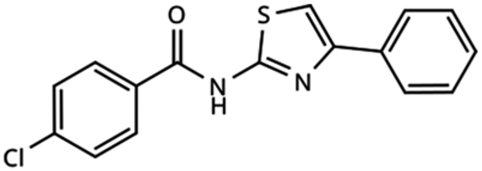
MV3011	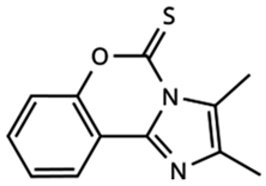	**8**	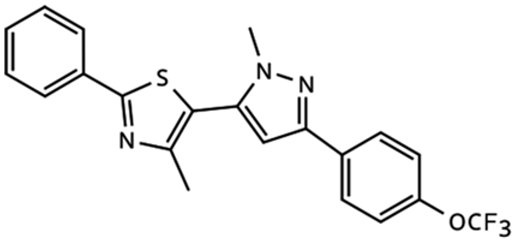

**Table 2:** Compounds **T1**–**T7** are the compounds designed in silico starting from MV3006, incorporating a fumarate hydrazide moiety at various positions and different substitutions deemed useful to improve the binding affinity. Molecular docking scores predicting the binding affinity of the ALKBH2 inhibitors evaluated computationally in this study are reported.

Compound	Structure	ALKBH2 Docking Score (kcal/mol)	ALKBH5 Docking Score (kcal/mol)	Compound	Structure	ALKBH2 Docking Score (kcal/mol)	ALKBH5 Docking Score (kcal/mol)
**T1**	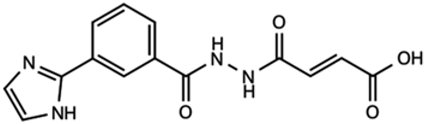	−14.8	−14.6	**T5**	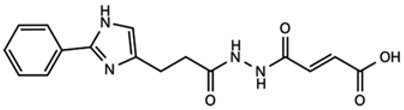	−14.9	−15.3
**T2**	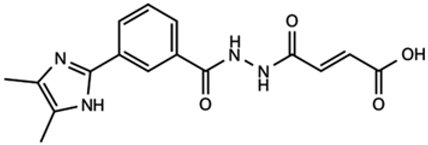	−14.9	−15.7	**T6**	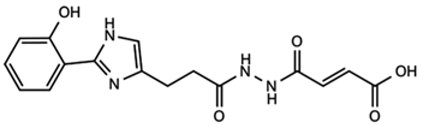	−15.1	−14.7
**T3**	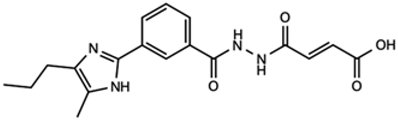	−15.3	−15.0	**T7**	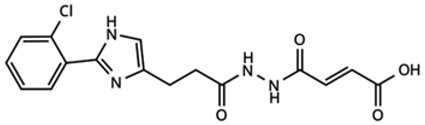	−14.2	−14.7
**T4**	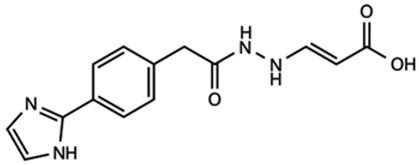	−15.4	−14.8				

**Table 3:** Structures of synthesized compounds.

Compound	Formula	Compound	Formula
**1**	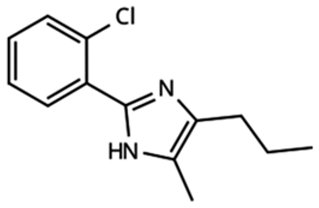	**9**	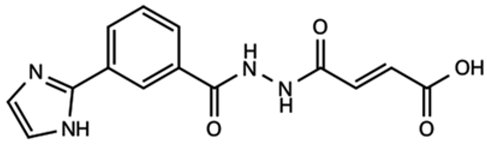
**2**	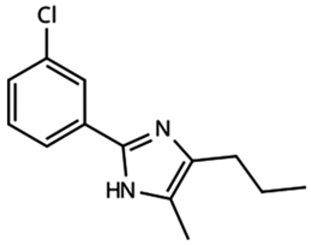	**10**	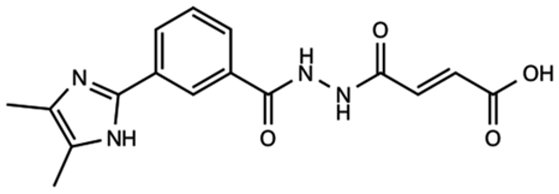
**3**	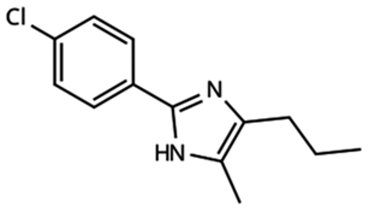	**11**	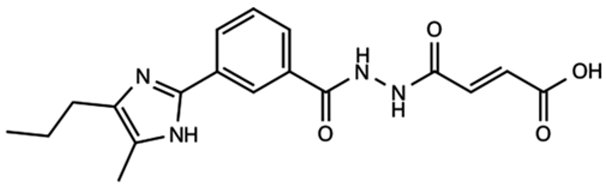
**4**	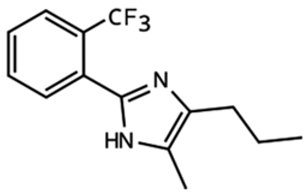	**12**	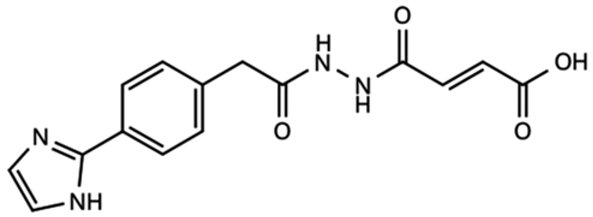
**5**	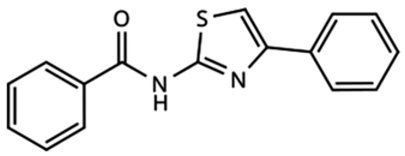	**13**	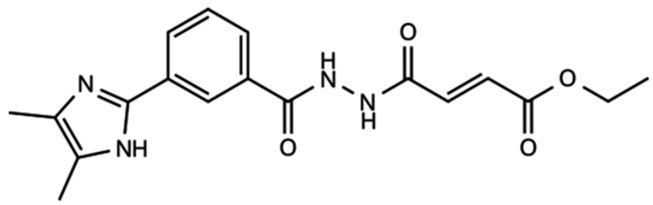
**6**	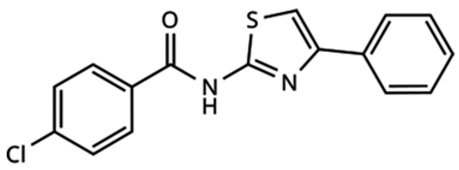	**14**	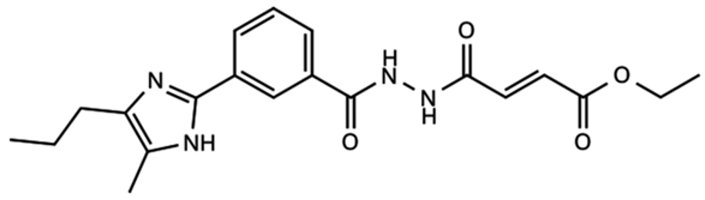
**7**	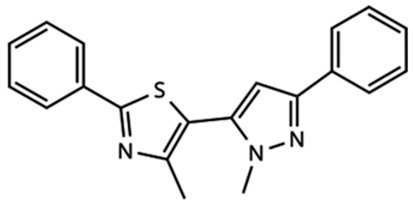	**15**	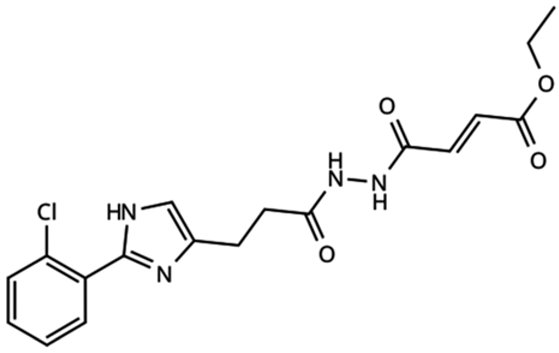
**8**	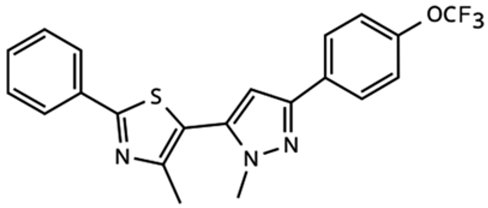		

**Table 4:** IC50 of TMZ in combination with different concentrations of MV3030 against U87-MG, G179, GSC7 and astrocyte cells.

TMZ IC50 µM	MV3030			
	0 µM	10 µM	25 µM	50 µM
U87-MG	>1200	>1200	>1200	1107 ± 86
G179	>1200	>1200	1033 ± 27	557 ± 58
GSC7	>1200	>1200	907 ± 22	512 ± 93
Astrocytes	>1200	>1200	>1200	1039 ± 65

## Data Availability

The raw data supporting the conclusions of this article will be made available by the authors on request.

## Supplementary Materials

The following supporting information can be downloaded at: https://www.mdpi.com/article/10.3390/biology15161371/s1, S1—Schemes and synthesis for target compounds 1–15. Figure S1. Cell viability of U87-MG cells, treated with MV1035 or compounds 1–15 (50 μM) for 48 and 72 h. Graph represents mean percentage ± SD of cell viability compared to untreated controls, arbitrarily set to 100%. ** *p* < 0.01 vs. CTRL; °° *p* < 0.01 vs. MV1035. Figure S2. Drug synergy and interaction analysis in G179 cells. Bliss (A) and ZIP (B) synergy heatmaps showing a predominantly synergistic profile. (C) Chou-Talalai Fraction Affected (FA)–Combination Index (CI) plot. The green data point below the dashed threshold line (CI = 1.0) indicates the synergistic combinations. (D) Summary table of drug combinations, highlighting the highly synergistic treatment window (green) and the strongly antagonistic combination (red). Figure S3. Drug synergy and interaction analysis in GSC7 cells. Bliss (A) and ZIP (B) synergy heatmaps showing a predominantly synergistic profile. (C) Chou-Talalai Fraction Affected (FA)–Combination Index (CI) plot. The green data point below the dashed threshold line (CI = 1.0) indicates the synergistic combinations. (D) Summary table of drug combinations, highlighting the highly synergistic treatment window (green) and the strongly antagonistic combination (red). Figure S4. Drug synergy and interaction analysis in U87-MG cells. Bliss (A) and ZIP (B) synergy heatmaps showing a predominantly additive profile. (C) Chou-Talalai Fraction Affected (FA)–Combination Index (CI) plot. The green data point below the dashed threshold line (CI = 1.0) indicates the synergistic combinations. (D) Summary table of drug combinations, highlighting the highly synergistic treatment window (green) and the strongly antagonistic combination (red). Figure S5. Drug synergyand interaction analysis in astrocytes. Bliss (A) and ZIP (B) synergy heatmaps showing a predominantly additive profile. (C) Chou-Talalai Fraction Affected (FA)–Combination Index (CI) plot. The green data point below the dashed threshold line (CI = 1.0) indicates the synergistic combinations. (D) Summary table of drug combinations, highlighting the highly synergistic treatment window (green) and the strongly antagonistic combination (red). Figure S6. Comparative analysis of glioblastoma and healthy astrocyte responses to treatments. Cell viability dose-response curves were compared across all glioblastoma cell lines used and healthy human astrocytes following 24 h of treatment. *p*-values were determined using the Extra sum-of-squares F-test. Synergistic interaction between TMZ and MV3030 is also shown for each cell type; Δ Score represents the difference in synergy score between glioblastoma and healthy cells. Green indicates values where healthy astrocytes demonstrate significantly higher resistance compared to glioblastoma cells, whereas red highlights values suggesting a potential inhibition of drug efficacy. Figure S7. Representative images of neurosphere in GSC7 (upper panel) and G179 (lower panel) cells following 7 days of treatment. The conditions shown include DMSO control, Temozolomide (TMZ), MV1035, MV3030, TMZ + MV1035, and TMZ + MV3030. Images were acquired from wells seeded with the highest cell density (3200 cells for GSC7 and 1200 cells for G179), as these best reflected the effects observed after 7 days of pharmacological treatment. All images were captured using a Nikon Eclipse microscope Ts2 at 10x magnification. Neurospheres are clearly visible in the DMSO control wells, while treatment with individual compounds resulted in a noticeable reduction in neurosphere size. In the combination treatments, only isolated dying cells remained, indicating a strong inhibitory effect on neurosphere formation. Figure S8. FoxM1, β-Catenin, Cyclin E and Survivin expression modulation in U87-MG cells. Cells were treated with MV1035 (50 µM), MV3030 (50 μM), TMZ (600 µM) and their combinations for 24 h. Representative images of immunoblotting of FoxM1 (A), β-Catenin (B), Cyclin E (C) and Survivin (D), their respective actin and the graph representing the mean percentage ± SD of protein expression compared to untreated controls, arbitrarily set to 100 %. ** *p* < 0.01 vs. CTRL; °° *p* < 0.01 vs. MV1035. Figure S9. FoxM1 and β-Catenin immunofluorescence in U87-MG cells. U87-MG cells were treated with MV1035 (50 µM), MV3030 (50 μM), TMZ (600 µM) and their combinations for 24 h. Samples were immunostained for FoxM1 (green) and β-catenin (red). Nuclei were counterstained with DAPI (blue). Figure S10. Cell free ALKBH2 (A) and ALKBH5 (B) demethylase activity assay in presence of different concentrations of MV1035 or MV3030. Data are represented as mean percentage ± SD compared to untreated controls, arbitrarily set to 100 %. The Extra sum-of-squares F-test was used to calculate the *p*-value. Figure S11. Structural comparison of the predicted binding modes of the active fumarate hydrazide derivative within the ALKBH2 and ALKBH5 catalytic pockets. In the ALKBH2 active site (left), the phenyl-imidazole scaffold is optimally stabilized by π-π stacking interactions with residue Phe124. The ligand is deeply anchored by the distal fumarate hydrazide chain coordinating the catalytic metal ion (purple sphere). In the ALKBH5 active site (right), the corresponding residue Tyr141 exhibits a slightly shifted aromatic plane, influenced by its local environment and neighboring residues such as Arg130 and Asp125. Table S1. Predicted molecular docking scores for the initial set of compounds (MV1035, MV3003-MV3012, and 1–8) against ALKBH2 and ALKBH5 active sites. Table S2. Authentication of the glioma stem cell (GSC) lines through short tandem repeat (STR) genotyping. The numerical value and the locus of each marker are reported. Regarding G179, in order to demonstrate the absence of cross-contamination and the reliability of the cell line, a comparison between the STR profiles of two different batches, the one used in Baronchelli’s work (#) [20] and the one used in the current article (*), was performed. For GSC7, the STR profiles of the original tumor biopsy DNA (BP7) and the derived cell line were compared.

## Abbreviations

The following abbreviations are used in this manuscript:

α-KG	α-Ketoglutarate
BBB	Blood–Brain Barrier
CNS	Central Nervous System
CNS-MPO	Central Nervous System—Multiparameter Optimization
ELDA	Extreme Limiting Dilution Assay
EP	Endothelial Permeability
GBM	Glioblastoma Multiforme
GSC	Glioma Stem Cell
LDA	Limiting Dilution Assay
MTT	[3-(4,5-dimethylthiazol-2-yl)-2,5-diphenyltetrazolium bromide]
PD	Patient-Derived
RT	Room Temperature
SD	Standard Deviation
TEER	Transendothelial Electrical Resistance
TMZ	Temozolomide
ZIP	Zero Interaction Potency
