# Supplementary material for: Design and Biological Evaluation of ALKBH2 and ALKBH5 Inhibitors as Adjuvants to Temozolomide-Based Glioblastoma Treatment
Source: Biology (Basel). 2026 Aug 12;15(16):1371. doi: 10.3390/biology15161371 (PMC13510024; doi:10.3390/biology15161371)
Supplement: Supplementary file 1 [file biology-15-01371-s001.zip › biology-4447195-supplementary.pdf]

Supplementary Materials

Article

Design and Biological Evaluation of ALKBH2 and ALKBH5 Inhibitors as Adjuvants to Temozolomide-based Glioblastoma Treatment

Mirko Rivara [*,1], Alessio Malacrida [*,2], Martina Ghizzi [2,3], Angela Bentivegna [2,4,5,6], Francesco Saverio Sica [2], Francesca Re [2,4,5,6], Stefano Motta [6,7], Lara Callea [8], Laura Bonati [8], Matteo Incerti [1], Valentina Zuliani [1], Gabriella Nicolini [2,5,6].

*Authors equally contributed to the manuscript

[1] Food and Drug Department, University of Parma, Parco Area delle Scienze 27/A, 43124, Parma, PR, Italy

[2] School of Medicine and Surgery, Experimental Neurology Unit, University of Milano-Bicocca, Via Cadore 48, 20900, Monza, MB, Italy

[3] PhD Program in Neuroscience, School of Medicine and Surgery, University of Milano-Bicocca, 20900, Monza, Italy

[4] Fondazione IRCCS San Gerardo dei Tintori, 20900, Monza, Italy

[5] GBM-BI-TRACE (GlioBlastoMa-BIcocca-TRAnslational-CEnter), University of Milano-Bicocca, 20900, Monza, Italy

[6] Inter-University Center for the Promotion of the 3Rs Principles in Teaching & Research (Centro 3R), 56122, Pisa, Italy

[7] Department of Earth and Environmental Sciences, University of Milano-Bicocca, Piazza della Scienza 1, 20126 Milan, Italy

Corresponding author: Alessio Malacrida – alessio.malacrida@unimib.it

S1 - Schemes and synthesis for target compounds 1-15

Microwave assisted synthesis was performed using a CEM Microwave Synthesizer—Discover Model 2.0. The final products were analyzed on a ThermoQuest (Italia) FlashEA 1112 Elemental Analyzer, for C, H, N. The percentages recorded were within ±0.4% of the theoretical values. All products were also characterized by ^{1}H NMR and ^{13}C NMR. The ^{1}H NMR and ^{13}C NMR spectra were recorded on a Bruker 400 Avance spectrometer (400 MHz); chemical shifts (δ scale) are reported in parts per million (ppm) relative to the central peak of the solvent. ^{1}H NMR spectra are reported in order: multiplicity and number of protons; signals were characterized as s (singlet), dd (doublet of doublet), t (triplet), m (multiplet). Mass spectra were recorded on a single quadrupole analyzer Adivion Expression-L Compact Mass Spectrometer.

General procedure for the synthesis of compounds 1-4

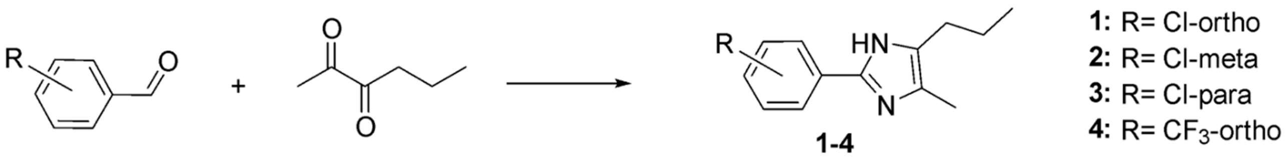

To a solution of benzaldehyde (1.00 mmol) in MeOH (3 mL) was added NH_4COOCH_3 (5.00 mmol) followed by the addition of a solution of hexane-2,3-dione (1.00 mmol) in MeOH (3 mL). The resulting mixture was stirred at room temperature (r.t.) overnight.

The methanol was then evaporated under reduced pressure and the residue quenched with a saturated aqueous solution of $NaHCO_3$ and extracted three times with EtOAc. The organic layers were collected and dried over anhydrous Na_2SO_4 and evaporated under reduced pressure.

The residue was purified by flash chromatography (SiO_2, DCM/EtOAc = 20:1). Compounds **1-4** were then crystallized as hydrochloride from abs EtOH and anhydrous Et_2O.

2-(2-chlorophenyl)-4-methyl-5-propyl-1H-imidazole hydrochloride (1). White solid; Yield: 67 %; ^{1}H NMR (400 MHz, DMSO-d_6) δ 7.95 (d, 1H), 7.63 (d, 1H),), 7.46 (m, 2H), 2.56 (m, 2H), 2.21 (s, 3H), 1.60 (m, 2H), 0.90 (t, 3H); ^{13}C NMR (100 MHz, DMSO-d_6) δ = 147.20, 135.09, 132.17, 130.22, 128.12, 126.42, 28.47, 21.97, 13.80, 10.89; APCI, [M + H] +): 235; C,H,N ($C_{13}H_{16}Cl_2N_2$): C_{calc} 57.58 %, C_{found} 57.39 %, H_{calc} 5.95 %, H_{found} 5.97 %, N_{calc} 10.33 %, N_{found} 10.43 %.

2-(3-chlorophenyl)-4-methyl-5-propyl-1H-imidazole hydrochloride (2). White solid; Yield: 71 %; ^{1}H NMR (400 MHz, DMSO-d_6) δ = 7.99 (s, 1H), 7.89 (d, 1H), 7.49 (m, 2H), 2.54 (m, 2H), 2.20 (s, 3H), 1.60 (m, 2H), 0.89 (t, 3H); ^{13}C NMR (100 MHz, DMSO-d_6) δ = 148.80, 136.79, 132.70, 129.62, 128.93, 125.42, 27.17, 22.67, 13.72, 10.29; APCI, [M + H] +): 235; C,H,N ($C_{13}H_{16}Cl_2N_2$): C_{calc} 57.58 %, C_{found} 57.70 %, H_{calc} 5.95 %, H_{found} 6.01 %, N_{calc} 10.33 %, N_{found} 10.22 %.

2-(4-chlorophenyl)-4-methyl-5-propyl-1H-imidazole hydrochloride (3). White solid; Yield: 62 %; ^{1}H NMR (400 MHz, DMSO-d_6) δ = 7.93 (d, 2H), 7.60 (d, 2H), 2.54 (m, 2H), 2.21 (s, 3H), 1.60 (m, 2H), 0.90 (t, 3H); ^{13}C NMR (100 MHz, DMSO-d_6) δ = 147.11, 137.34, 133.12, 132.03, 127.53, 126.09, 28.17, 22.01, 14.04, 10.56; APCI, [M + H] +): 235; C,H,N ($C_{13}H_{16}Cl_2N_2$): C_{calc} 57.58 %, C_{found} 57.85 %, H_{calc} 5.95 %, H_{found} 6.03 %, N_{calc} 10.33 %, N_{found} 10.12 %.

4-methyl-5-propyl-2-(2-(trifluoromethyl)phenyl)-1H-imidazole (4). White solid; Yield: 69 %; ^{1}H NMR (400 MHz, DMSO-d_6) δ 7.90 (d, 1H), 7.61 (d, 1H),), 7.42 (m, 2H), 2.55 (m, 2H), 2.25 (s, 3H), 1.63 (m, 2H), 0.90 (t, 3H); ^{13}C NMR (100 MHz, DMSO-d_6) δ = 148.20, 134.93, 133.02, 130.22, 129.61, 126.01, 28.10, 21.20, 13.10, 10.29; APCI, [M + H] +): 269; C,H,N ($C_{14}H_{16}ClF_3N_2$): C_{calc} 55.18 %, C_{found} 54.99 %, H_{calc} 5.29 %, H_{found} 5.40 %, N_{calc} 9.19 %, N_{found} 9.26 %.

General procedure for the synthesis of compounds 5, 6.

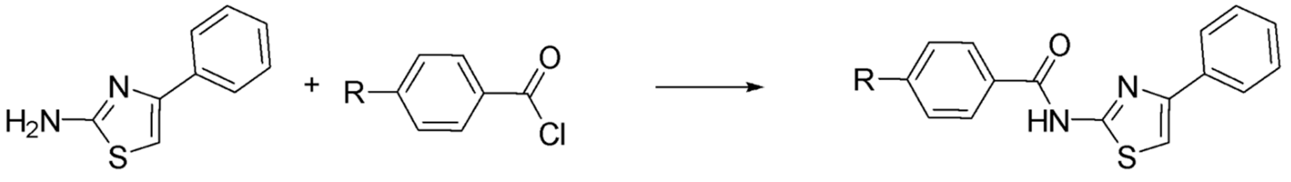

5: R= H
6: R= Cl

4-phenylthiazol-2-amine (1.0 mmol) was dissolved in 1.2 mL of triethylamine and 4.0 mL of THF and then benzoyl chloride (or 4-chloro benzoyl chloride) (1.0 mmol) was added dropwise maintaining the temperature between 0 and 5 °C. The reaction mixture was stirred for 5h at room temperature; after that it was neutralized with 10% HCl solution and a solid precipitated. The precipitate was washed with water, dried and then recrystallized from abs EtOH.

N-(4-phenylthiazol-2-yl)benzamide (5). yellow solid; Yield: 59 %; ^{1}H NMR (400 MHz, DMSO-d_6) δ = 8.14 (d, 2H), 7.96 (d, 2H), 7.70 (s, 1H), 7.43-7.66 (m, 5H), 7.33 (t, 1H); ^{13}C NMR (100 MHz, DMSO-d_6) δ = 165.75, 159.01, 149.61, 134.82, 133.35, 133.12, 129.73, 129.23, 129.08, 128.67, 128.3, 126.26; APCI, [M + H] +): 281; C,H,N ($C_{16}H_{12}N_2OS$): C_{calc} 68.55 %, C_{found} 68.37 %, H_{calc} 4.31 %, H_{found} 4.44 %, N_{calc} 9.99 %, N_{found} 10.11 %.

4-chloro-N-(4-phenylthiazol-2-yl)benzamide (6). White solid; Yield: 78 %; ^{1}H NMR (400 MHz, DMSO-d_6) δ = 8.14 (d, 2H), 7.96 (d, 2H), 7.72 (s, 1H), 7.64 (d, 2H), 7.46 (t, 2H), 7.34 (t, 1H); ^{13}C NMR (100 MHz, DMSO-d_6) δ = 166.95, 160.91, 149.89, 135.12, 132.88, 131.22, 129.63, 129.03, 129.01, 128.02, 127.84, 124.56; APCI, [M + H] +): 315; C,H,N ($C_{16}H_{11}ClN_2OS$): C_{calc} 61.05 %, C_{found} 60.79 %, H_{calc} 3.52 %, H_{found} 3.66 %, N_{calc} 8.90 %, N_{found} 9.05 %.

General procedure for the synthesis of compounds 7, 8.

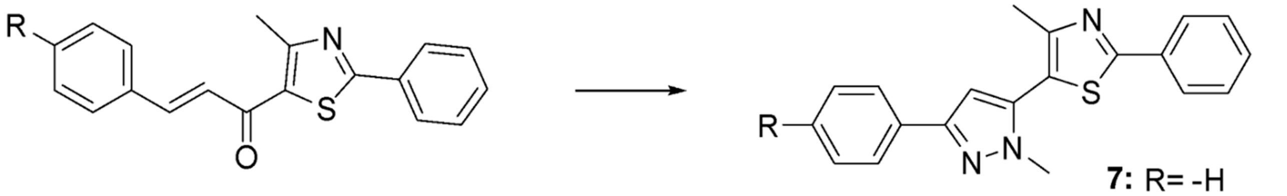

7: R= -H
8: R= -OCF3

A mixture of chalcone (1.71 mmol) in anhydrous ethanol (25 mL) under nitrogen was heated to reflux until the solid was completely dissolved, then 3.59 mmol of 2-methylhydrazine previously dissolved in 1 mL of anhydrous ethanol was added dropwise. The reaction mixture was heated to reflux for 5 hours. The solvent was then removed under reduced pressure obtaining a yellow solid which was purified by flash chromatography [SiO_2 Petroleum ether: ethyl acetate 90:10]

1-methyl-5-(4-methyl-2-phenylthyaazol-5-yl)-3-phenyl-1H-pyrazole (7). White solid; Yield: 22%; ^{1}H-NMR (400 MHz CDCl3) δ= 2.49 (s,3H); 3.91 (s,3H); 6.57 (s,1H); 7.44-7.51 (m,6H); 7.84-7.86 (m,2H); 7.87-7.90 (m,2H). ^{13}C-NMR (100 MHz $CDCl_3$) δ= 16.24; 37.39; 104.81; 118.93; 125.53; 126.51; 126.64; 128.94; 130.47; 132.95; 132.78; 134.65; 145.30; 150.83; 153.23; 165.01; APCI, [M + H] +): 332; C,H,N ($C_{20}H_{17}N_3S$): C_{calc} 72.48 %, C_{found} 72.69 %, H_{calc} 5.17 %, H_{found} 5.05 %, N_{calc} 12.68 %, N_{found} 12.22 %.

1-methyl-5-(4 methyl-2-phenylthialol-5-yl)-3y-(4-(trifluoromethoxy)phenyl)-1H-pyrazole (8). White solid; Yield: 23%; ^{1}H-NMR (400 MHz $CDCl_3$) δ= 2.48 (s,3H); 3.90 (s,3H); 6.68 (s,1H); 7.27-7.29 (m,2H); 7.47-7.52 (m,3H); 7.85-7.87 (m,2H); ; 7.96-7.99 (m,2H); ^{13}C-NMR (100 MHz CDCl3) δ= 16.21; 37.42; 105.09; 105.87; 118.66; 121.22; 121.30; 126.52; 126.66; 126.86; 129.09; 130.37; 130.53; 134.91; 148.86; 149.53; 153.32; 168.20; APCI, [M + H] +): 416; C,H,N ($C_{21}H_{16}F_3N_3OS$): C_{calc} 60.72 %, C_{found} 60.51 %, H_{calc} 3.88 %, H_{found} 3.99 %, N_{calc} 10.11 %, N_{found} 10.33 %.

General procedure for the synthesis of compounds 9-11.

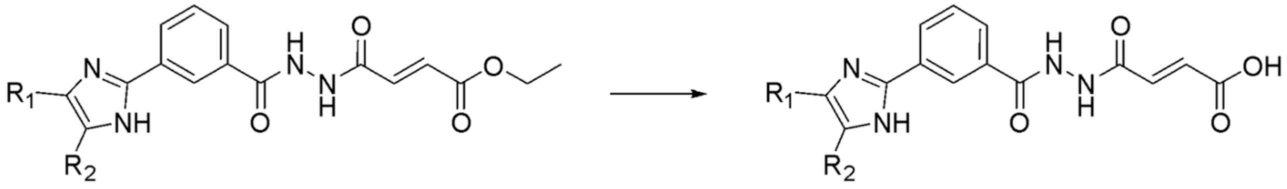

9: R_1= -H; R_2=-H
10: R_1= $-CH_3$; R_2=$-CH_3$
11: R_1= $-CH_3$; R_2=$-CH_2CH_2CH_3$

The proper ester (1.0 mmol) reacts with LiOH*H_2O (1.3 mmol) in 5.0 mL of a mixture H2O/THF (1:2) at room temperature overnight.
After completion the reaction mixture was acidified with diluted HCl, a solid was collected, dried and recrystallized from EtOH/EtO_2.

(E)-4-(2-(3-(1H-imidazol-2-yl)benzoyl)hydrazinyl)-4-oxobut-2-enoic acid (9). yellow solid; Yield: 59 %; ^{1}H NMR (400 MHz, DMSO-d_6) δ = 8.50 (s, 1H), 8.18 (t, 2H), 7.82 (t, 1H), 7.69 (s, 2H), 7.13 (d, 1H), 6.86 (d, 1H); ^{13}C NMR (100 MHz, DMSO-d_6) δ = 168.56, 167.23, 166.96, 147.55, 131.54, 131.06, 129.88, 127.92, 127.28, 126.06, 125.20; APCI, [M + H] +): 301; C,H,N ($C_{14}H_{12}N_4O_4$): C_{calc} 56.00 %, C_{found} 56.32 %, H_{calc} 4.03 %, H_{found} 4.09 %, N_{calc} 18.66 %, N_{found} 18.53 %.

(E)-4-(2-(3-(4,5-dimethyl-1H-imidazol-2-yl)benzoyl)hydrazinyl)-4-oxobut-2-enoic acid (10). white solid; Yield: 70 %; ^{1}H NMR (400 MHz, CD_3OD) δ = 8.45 (s, 1H), 8.13 (d, 2H), 7.80 (t, 1H), 7.13 (d, 1H), 6.86 (d, 1H), 2.38 (s, 6H); ^{13}C NMR (100 MHz, DMSO-d_6) δ = 167.08, 166.04, 165.88, 145.99, 133.11, 132.74, 130.21, 127.22, 126.85, 125.23, 124.44, 124.03, 10.11; APCI, [M + H] +): 329; C,H,N ($C_{16}H_{16}N_4O_4$): C_{calc} 58.53 %, C_{found} 58.71 %, H_{calc} 4.91 %, H_{found} 5.01 %, N_{calc} 17.06 %, N_{found} 17.22 %.

(E)-4-(2-(3-(5-methyl-4-propyl-1H-imidazol-2-yl)benzoyl)hydrazinyl)-4-oxobut-2-enoic acid (11). white solid; Yield: 73 %; ^{1}H NMR (400 MHz, DMSO-d_6) δ = 8.77 (s, 1H), 8.53 (d, 1H), 8.06 (d, 1H), 7.72 (t, 1H), 7.09 (d, 1H), 6.68 (d, 1H), 2.65 (t, 2H), 2.3 (s, 3H), 1.68 (m, 2H), 0.89 (t, 3H);^{13}C NMR (100 MHz, DMSO-d_6) δ = 168.11,

167.66, 166.50, 146.22, 134.05, 133.69, 131.21, 129.45, 127.56, 126.14, 125.33, 125.11, 28.99, 22.54, 14.58, 10.66; APCI, [M + H] +): 357; C,H,N ($C_{18}H_{20}N_4O_4$): C_{calc} 62.63 %, C_{found} 62.39 %, H_{calc} 5.28 %, H_{found} 5.15 %, N_{calc} 13.59 %, N_{found} 13.71 %.

General procedure for the synthesis of compound 12.

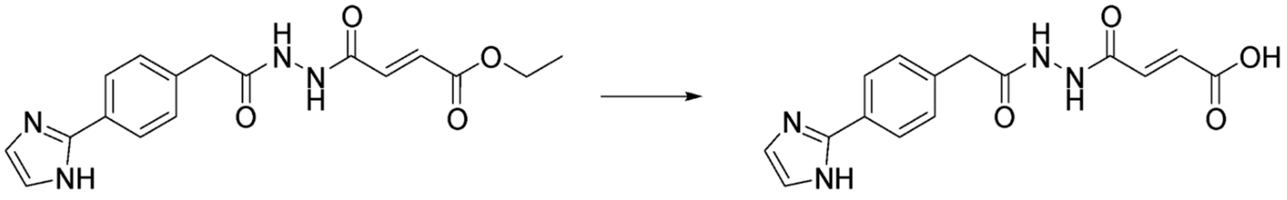

Ethyl (E)-4-(2-(2-(4-(1H-imidazol-2-yl)phenyl)acetyl)hydrazinyl)-4-oxobut-2-enoate (1.0 mmol) is reacted with LiOH*H2O (1.3 mmol) in 5.0 mL of a mixture H2O/THF (1:2) at room temperature overnight.
After completion the reaction mixture was acidified with diluted HCl, a solid was collected, dried and recrystallized from EtOH/EtO2.

(E)-4-(2-(2-(4-(1H-imidazol-2-yl)phenyl)acetyl)hydrazinyl)-4-oxobut-2-enoic acid (12). yellow solid; Yield: 59 %; ^{1}H NMR (400 MHz, CD_3OD) *δ* = 7.83 (d, 2H), 7.45 (d, 2H), 7.13 (s, 2H), 6.76 (s, 1H), 6.70 (s, 1H), 3.64 (s, 2H); ^{13}C NMR (100 MHz, CD_3OD) *δ* = 170.39, 167.96, 147.01, 133.55, 129.98, 128.69, 124.34, 99.77, 38.62; APCI, [M + H] +): 288; C,H,N ($C_{14}H_{14}N_4O_3$): C_{calc} 58.74 %, C_{found} 58.70 %, H_{calc} 4.93 %, H_{found} 5.11 %, N_{calc} 19.57 %, N_{found} 19.22 %.

General procedure for the synthesis of compounds 13 and 14.

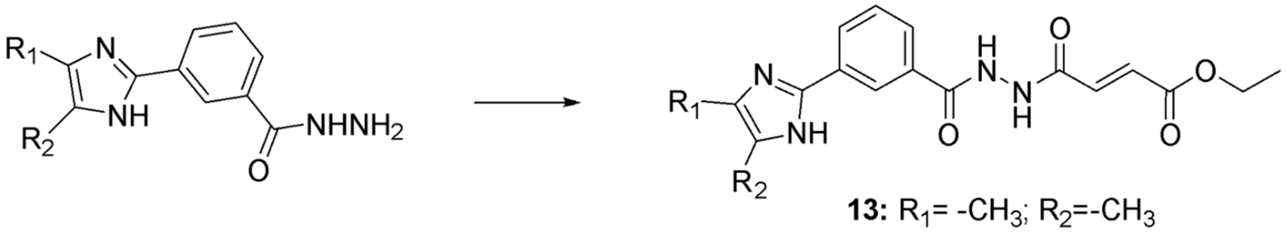

The proper benzo hydrazide (1.0 mmol) is reacted with perfluorophenyl (E)-4-oxohex-2-enoate (1.0 mmol)) in 5.0 mL of a mixture CH_3CN/DMSO (1:1) at room temperature overnight.
The desired products separate from the solution and are filtered and collected.

Ethyl (E)-4-(2-(3-(4,5-dimethyl-1H-imidazol-2-yl)benzoyl)hydrazinyl)-4-oxobut-2-enoate (13). yellow solid; Yield: 54 %; ^{1}H NMR (400 MHz, DMSO-*d*$_6$) *δ* = 8.36 (s, 1H), 8.02 (d, 1H), 7.75 (d, 1H), 7.53 (t, 1H), 7.12 (d, 1H), 6.73 (d, 1H), 4.03 (t, 2H), 2.14 (s, 6H), 1.20 (t, 3H)^{13}C NMR (100 MHz, DMSO-*d*$_6$) *δ* = 168.11, 166.06, 164.50, 146.87, 132.11, 131.95, 131.63, 129.45, 128.66, 128.14, 125.78, 125.65, 60.84, 14.54, 11.01, 11.63; APCI, [M + H] +): 357; C,H,N ($C_{18}H_{20}$ N_4O_4): C_{calc} 60.66 %, C_{found} 60.84 %, H_{calc} 5.66 %, H_{found} 5.97 %, N_{calc} 15.72 %, N_{found} 15.56 %.
Ethyl (E)-4-(2-(3-(5-methyl-4-propyl-1H-imidazol-2-yl)benzoyl)hydrazinyl)-4-oxobut-2-enoate (14). yellow solid; Yield: 48 %; ^{1}H NMR (400 MHz, DMSO-*d*$_6$) *δ* = 8.37 (s, 1H), 8.03 (d, 1H), 7.75 (d, 1H), 7.52 (t, 1H), 7.12 (d, 1H), 6.73 (d, 1H), 4.20 (t, 2H), 2.14 (s, 3H), 1.59 (m, 2H); 1.24 (t, 3H); 0.90 (t, 3H);^{13}C NMR (100 MHz, DMSO-*d*$_6$) *δ* = 166.61, 164.13, 148.88, 137.95, 133.71, 130.56, 130.23, 129.97, 128.32, 128.01, 126.68, 126.33, 60.21, 28.12, 21.80

14.64, 14.01, 11.63 APCI, [M + H] +): 385; C,H,N ($C_{20}H_{24}N_4O_4$): C_{calc} 62.49 %, C_{found} 62.26 %, H_{calc} 6.29 %, H_{found} 5.99 %, N_{calc} 14.57 %, N_{found} 14.33 %.

General procedure for the synthesis of compound 15.

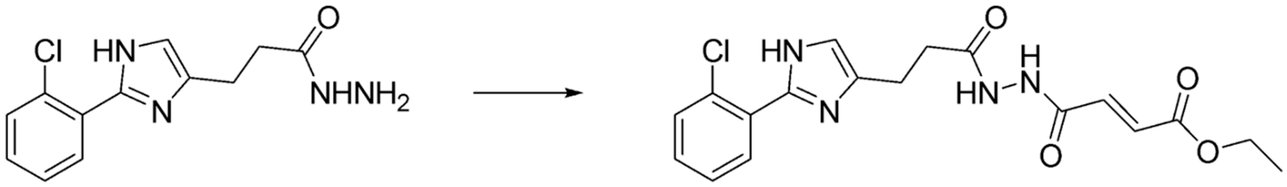

The 3-(2-(2-chlorophenyl)-1H-imidazol-4-yl)propanehydrazide (1.0 mmol) is reacted with perfluorophenyl (E)-4-oxohex-2-enoate (1.0 mmol) in 5.0 mL of a mixture CH3CN/DMSO (1:1) at room temperature overnight. The desired product separates from the solution and is filtered and collected.

Ethyl (E)-4-(2-(3-(2-(2-chlorophenyl)-1H-imidazol-4-yl)propanoyl)hydrazinyl)-4-oxobut-2-enoate (15). White solid; Yield: 33 %; ^{1}H NMR (400 MHz, DMSO-*d*$_6$) δ = 7.72 (d, 1H), 7.53 (d, 1H), 7.41 (m, 2H), 7.05 (d, 1H), 6.71 (d, 1H), 3.02 (t, 2H), 2.68 (t, 2H), 2.39 (t, 2H), 0.92 (t, 3H); ^{13}C NMR (100 MHz, DMSO-*d*$_6$) δ = 174.48, 165.26, 163.10, 131.70, 130.97, 130.35, 129.94, 129.86, 129.64, 126.83, 60.94, 36.47, 29.08, 24.98, 13.03; APCI, [M + H] +): 391; C,H,N ($C_{18}H_{19}Cl N_4O_4$): C_{calc} 55.32 %, C_{found} 55.11 %, H_{calc} 4.90 %, H_{found} 4.85 %, N_{calc} 14.34 %, N_{found} 14.59 %.

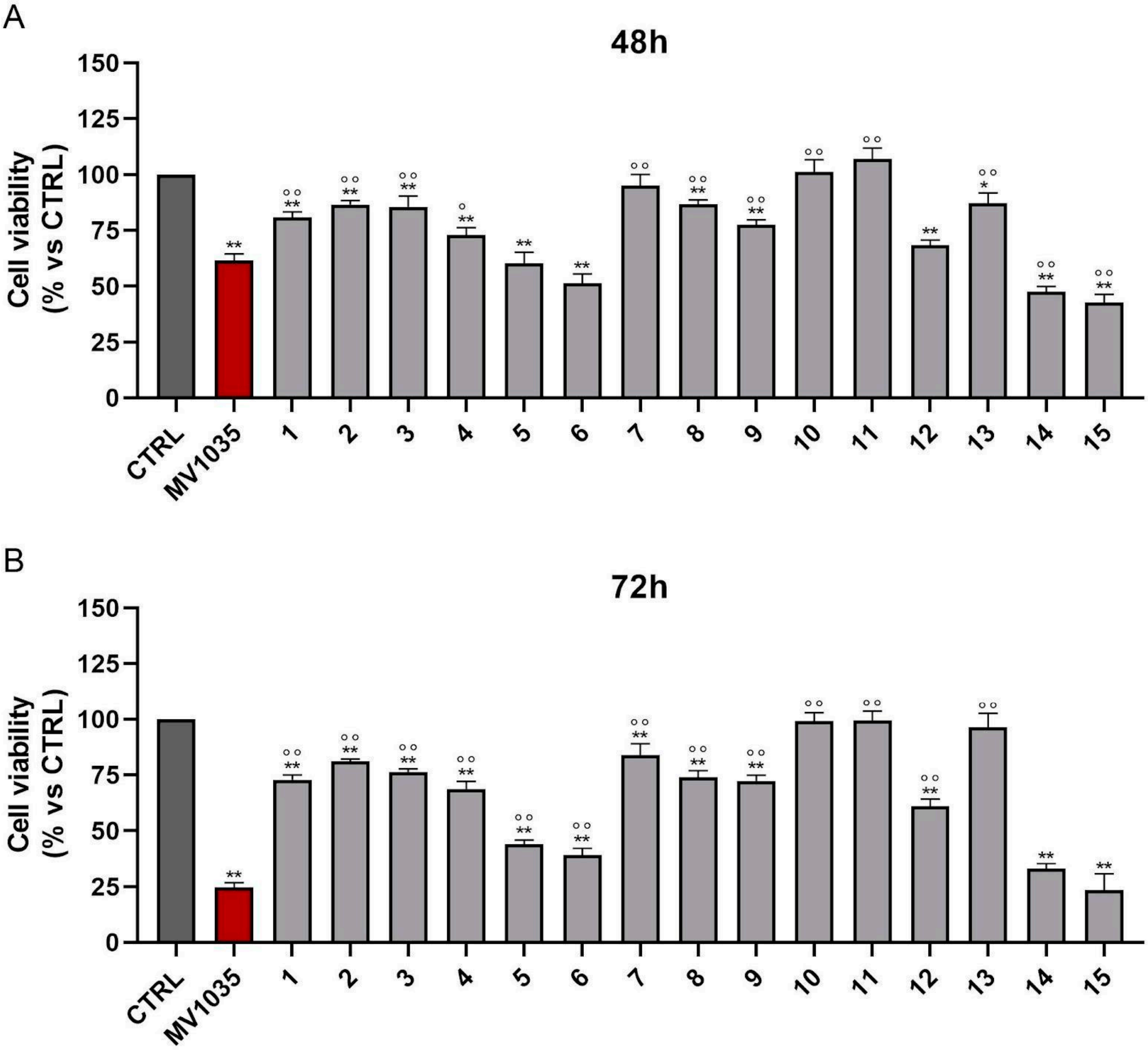

Supplementary Figure S1. Cell viability of U87-MG cells, treated with MV1035 or compounds 1-15 (50μM) for 48 and 72h. Graph represents mean percentage ± SD of cell viability compared to untreated controls, arbitrarily set to 100 %. **$p < 0.01$ vs. CTRL; °°$p < 0.01$ vs. MV1035.

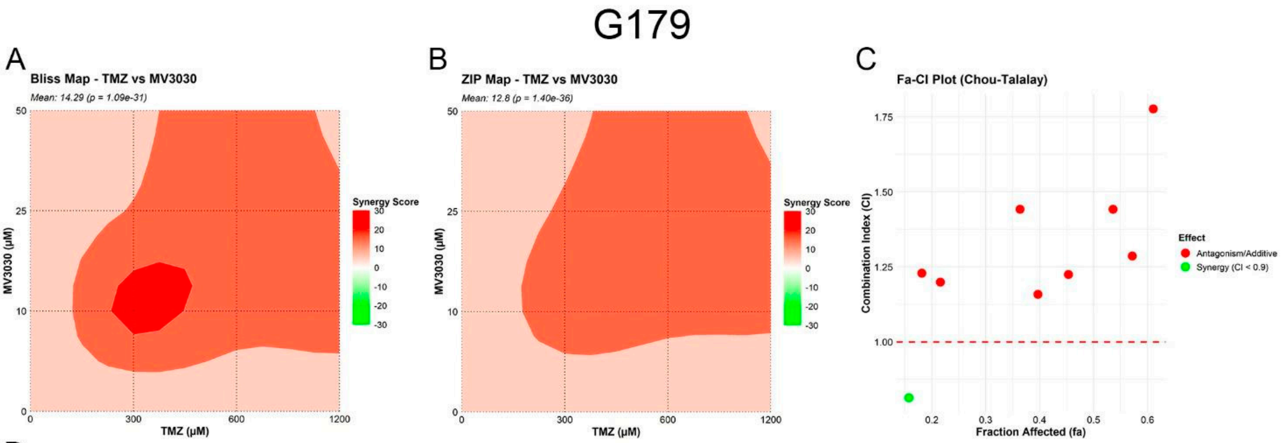

D

[TMZ] µM	[MV3030] µM	Single combination Bliss score	Single combination ZIP score	Chou-Talalai Combination Index (CI)	Chou-Talalai interpretation
300	10	24.72	13.61	0.81	Synergy
300	25	10.76	11.55	1.20	Moderate antag.
300	50	6.23	5.66	1.44	Antagonism
600	10	14.34	12.83	1.23	Antagonism
600	25	17.72	17.83	1.16	Moderate antag.
600	50	19.75	19.62	1.29	Antagonism
1200	10	16.41	12.30	1.22	Antagonism
1200	25	12.06	12.12	1.44	Antagonism
1200	50	6.62	6.70	1.78	Antagonism

Supplementary Figure S2. Drug synergy and interaction analysis in G179 cells. Bliss (A) and ZIP (B) synergy heatmaps showing a predominantly synergistic profile. (C) Chou-Talalai Fraction Affected (FA) – Combination Index (CI) plot. The green data point below the dashed threshold line (CI = 1.0) indicates the synergistic combinations. (D) Summary table of drug combinations, highlighting the highly synergistic treatment window (green) and the strongly antagonistic combination (red).

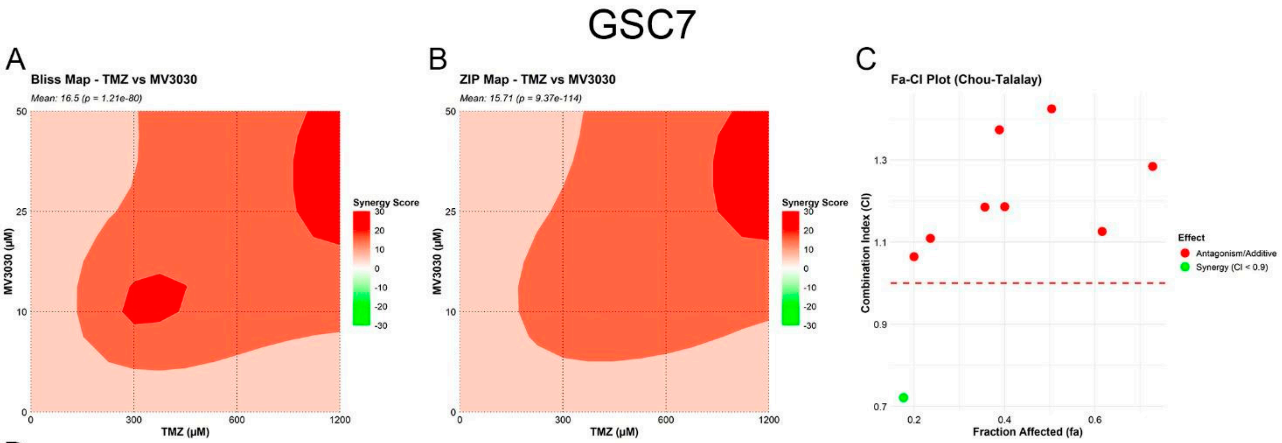

D

[TMZ] µM	[MV3030] µM	Single combination Bliss score	Single combination ZIP score	Chou-Talalai Combination Index (CI)	Chou-Talalai interpretation
300	10	22.30	17.46	0.72	Synergy
300	25	11.53	11.23	1.11	Moderate antag.
300	50	9.67	8.10	1.37	Antagonism
600	10	16.06	16.41	1.06	Additive
600	25	15.78	16.65	1.19	Moderate antag.
600	50	17.06	17.17	1.42	Antagonism
1200	10	12.51	10.97	1.19	Moderate antag.
1200	25	22.68	22.59	1.13	Moderate antag.
1200	50	20.87	20.81	1.28	Antagonism

Supplementary Figure S3. Drug synergy and interaction analysis in GSC7 cells. Bliss (A) and ZIP (B) synergy heatmaps showing a predominantly synergistic profile. (C) Chou-Talalai Fraction Affected (FA) – Combination Index (CI) plot. The green data point below the dashed threshold line (CI = 1.0) indicates the synergistic combinations. (D) Summary table of drug combinations, highlighting the highly synergistic treatment window (green) and the strongly antagonistic combination (red).

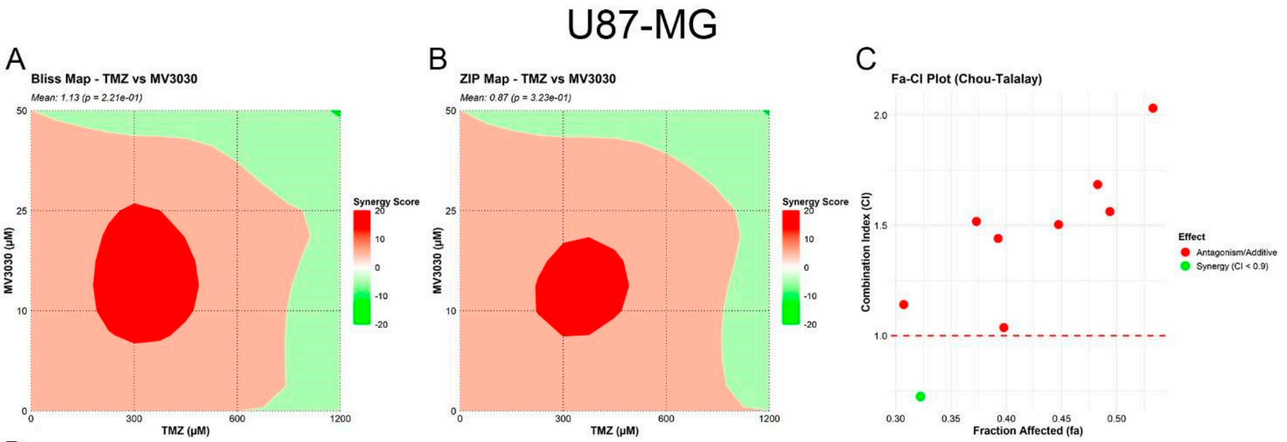

D

[TMZ] µM	[MV3030] µM	Single combination Bliss score	Single combination ZIP score	Chou-Talalai Combination Index (CI)	Chou-Talalai interpretation
300	10	14.60	12.87	0.72	Synergy
300	25	11.07	7.67	1.04	Additive
300	50	-3.32	-2.55	1.50	Antagonism
600	10	5.10	6.22	1.14	Moderate antag.
600	25	2.79	4.97	1.44	Antagonism
600	50	-3.69	-3.87	1.69	Antagonism
1200	10	-4.86	-4.97	1.52	Antagonism
1200	25	-0.83	-2.09	1.56	Antagonism
1200	50	-10.71	-10.47	2.03	Antagonism

Supplementary Figure S4. Drug synergy and interaction analysis in U87-MG cells. Bliss (A) and ZIP (B) synergy heatmaps showing a predominantly additive profile. (C) Chou-Talalai Fraction Affected (FA) – Combination Index (CI) plot. The green data point below the dashed threshold line (CI = 1.0) indicates the synergistic combinations. (D) Summary table of drug combinations, highlighting the highly synergistic treatment window (green) and the strongly antagonistic combination (red).

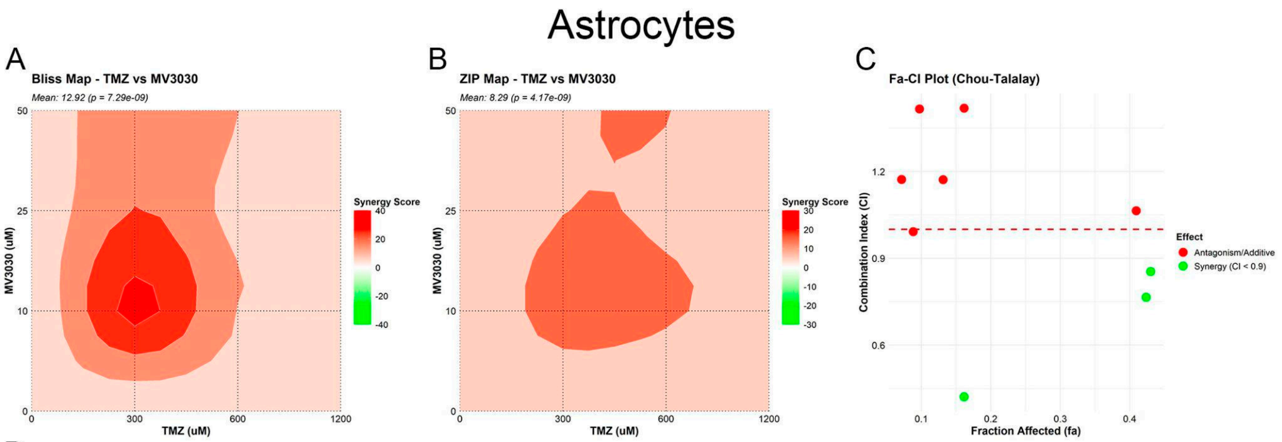

D

[TMZ] µM	[MV3030] µM	Single combination Bliss score	Single combination ZIP score	Chou-Talalai Combination Index (CI)	Chou-Talalai interpretation
300	10	34.43	15.19	0.42	Synergy
300	25	20.32	9.80	0.99	Additive
300	50	20.00	8.43	1.17	Moderate antag.
600	10	9.75	11.31	1.17	Moderate antag.
600	25	5.58	7.64	1.42	Antagonism
600	50	10.20	10.46	1.42	Antagonism
1200	10	9.41	6.83	0.77	Synergy
1200	25	6.19	4.84	0.85	Synergy
1200	50	0.37	0.12	1.06	Additive

Supplementary Figure S5. Drug synergyand interaction analysis in astrocytes. Bliss (A) and ZIP (B) synergy heatmaps showing a predominantly additive profile. (C) Chou-Talalai Fraction Affected (FA) – Combination Index (CI) plot. The green data point below the dashed threshold line (CI = 1.0) indicates the synergistic combinations. (D) Summary table of drug combinations, highlighting the highly synergistic treatment window (green) and the strongly antagonistic combination (red).

	Astrocytes		G179		GSC7		U87-MG	
	IC50		IC50	p	IC50	p	IC50	p
MV3030 0 µM + TMZ	1405		1925	0.2302	1986	0.2380	2379	0.1102
MV3030 10 µM + TMZ	1438		1388	0.8426	1742	0.3688	1887	0.1546
MV3030 25 µM + TMZ	2090		1033	0.0001	907	0.0111	1746	0.0001
MV3030 50 µM + TMZ	1039		557	0.0001	512.9	0.0058	1107	0.9872
MV3030	132.6		78.19	0.3117	92.00	0.4885	66.09	0.1911
	Score		Score	Δ Score	Score	Δ Score	Score	Δ Score
Bliss	12.92		14.29	1.37	16.50	3.58	1.13	-11.79
ZIP	8.29		12.80	4.51	15.71	7.42	0.87	-7.42

Supplementary Figure S6. Comparative analysis of glioblastoma and healthy astrocyte responses to treatments. Cell viability dose-response curves were compared across all glioblastoma cell lines used and healthy human astrocytes following 24 hours of treatment. p-values were determined using the Extra sum-of-squares F-test. Synergistic interaction between TMZ and MV3030 is also shown for each cell type; Δ Score represents the difference in synergy score between glioblastoma and healthy cells. Green indicates values where healthy astrocytes demonstrate significantly higher resistance compared to glioblastoma cells, whereas red highlights values suggesting a potential inhibition of drug efficacy.

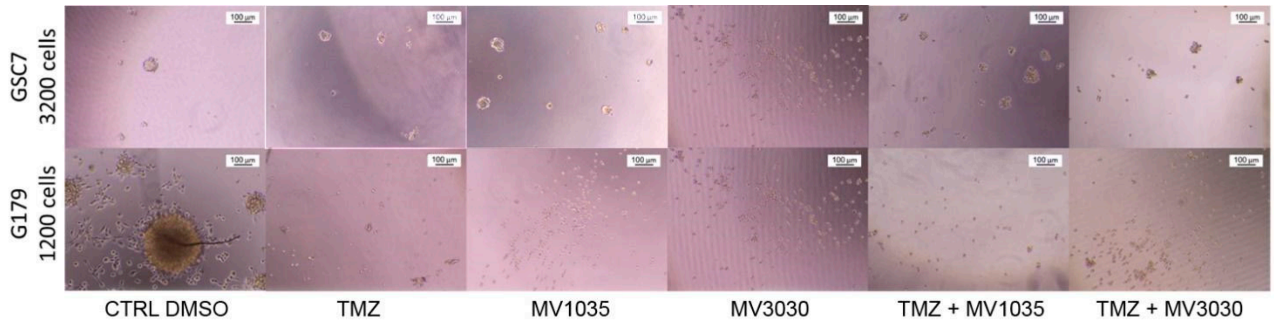

Supplementary Figure S7. Representative images of neurosphere in GSC7 (upper panel) and G179 (lower panel) cells following 7 days of treatment. The conditions shown include DMSO control, Temozolomide (TMZ), MV1035, MV3030, TMZ+MV1035, and TMZ+MV3030. Images were acquired from wells seeded with the highest cell density (3200 cells for GSC7 and 1200 cells for G179), as these best reflected the effects observed after 7 days of pharmacological treatment. All images were captured using a Nikon Eclipse microscope Ts2 at 10x magnification. Neurospheres are clearly visible in the DMSO control wells, while treatment with individual compounds resulted in a noticeable reduction in neurosphere size. In the combination treatments, only isolated dying cells remained, indicating a strong inhibitory effect on neurosphere formation.

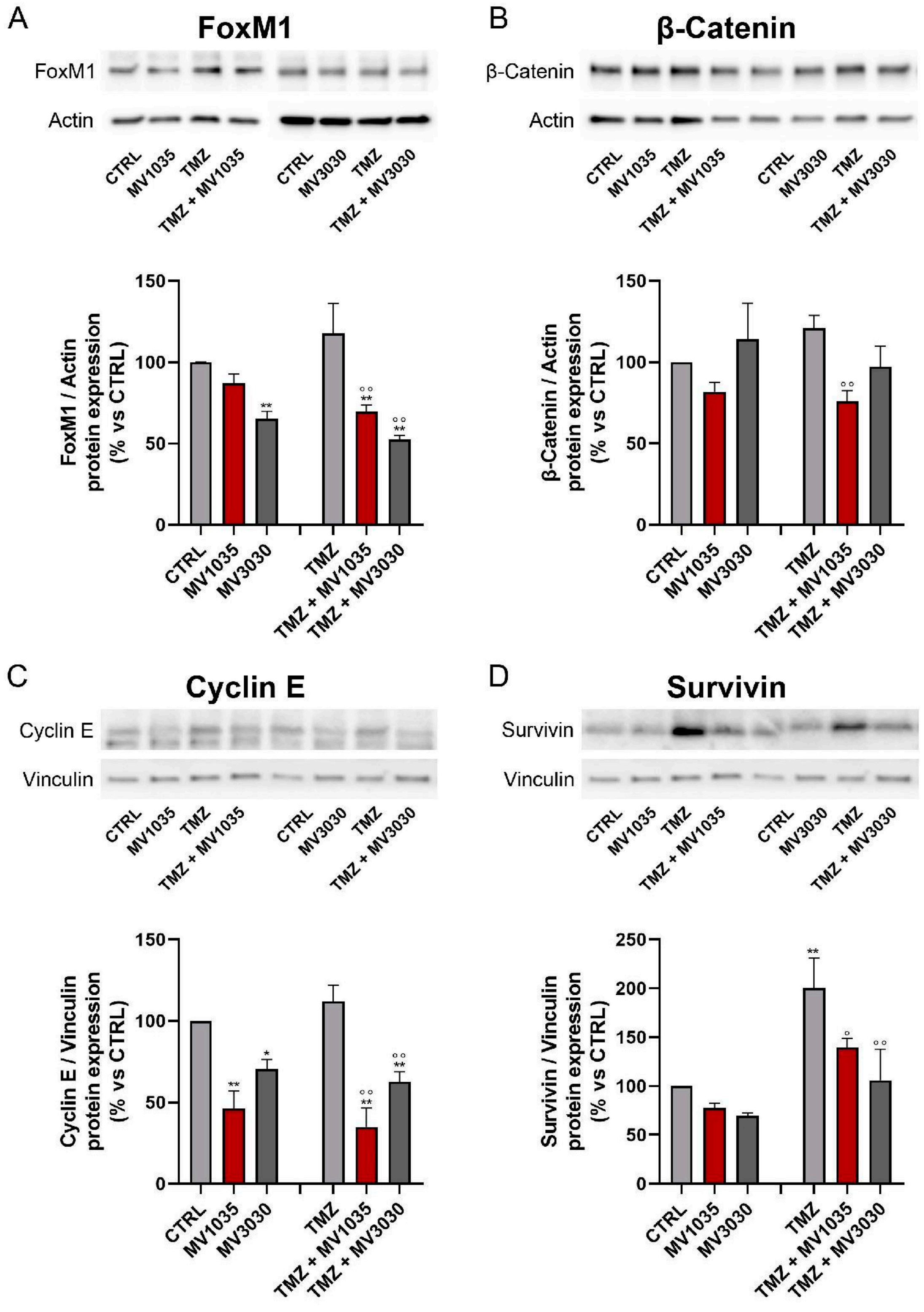

Supplementary Figure S8. FoxM1, β-Catenin, Cyclin E and Survivin expression modulation in U87-MG cells. Cells were treated with MV1035 (50μM), MV3030 (50μM), TMZ (600μM) and their combinations for 24 hours. Representative images of immunoblotting of FoxM1 (**A**), β-Catenin (**B**), Cyclin E (**C**) and Survivin (**D**), their respective actin and the graph representing the mean percentage ± SD of protein expression compared to untreated controls, arbitrarily set to 100 %. ** $p < 0.01$ vs. CTRL; °°$p < 0.01$ vs. MV1035. Blots in (**C-D**) were processed using one membrane per experimental group (MV1035 and MV3030), cut and probed for Cyclin E, Survivin, and Vinculin. All antibody specificities were previously validated prior to membrane cut.

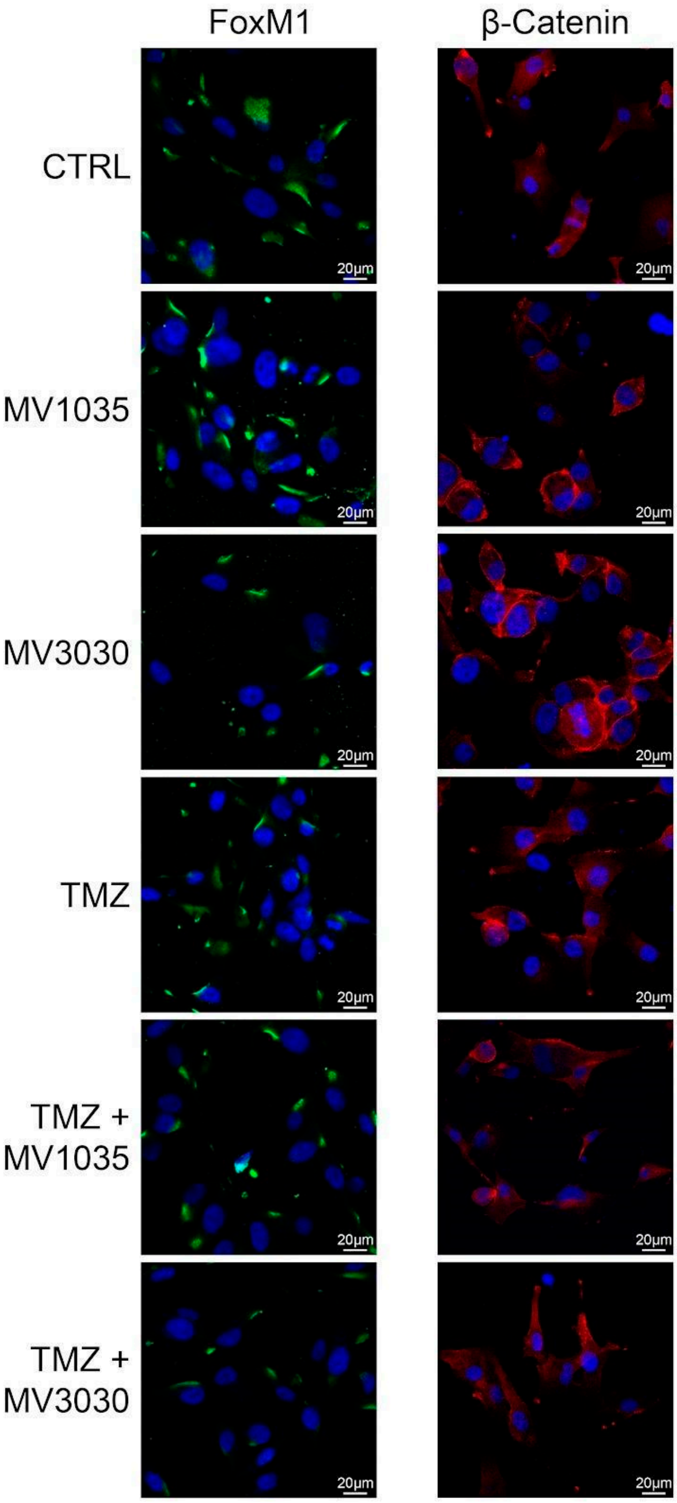

Supplementary Figure S9. FoxM1 and β-Catenin immunofluorescence in U87-MG cells. U87-MG cells were treated with MV1035 (50µM), MV3030 (50µM), TMZ (600µM) and their combinations for 24 hours. Samples were immunostained for FoxM1 (green) and β-catenin (red). Nuclei were counterstained with DAPI (blue).

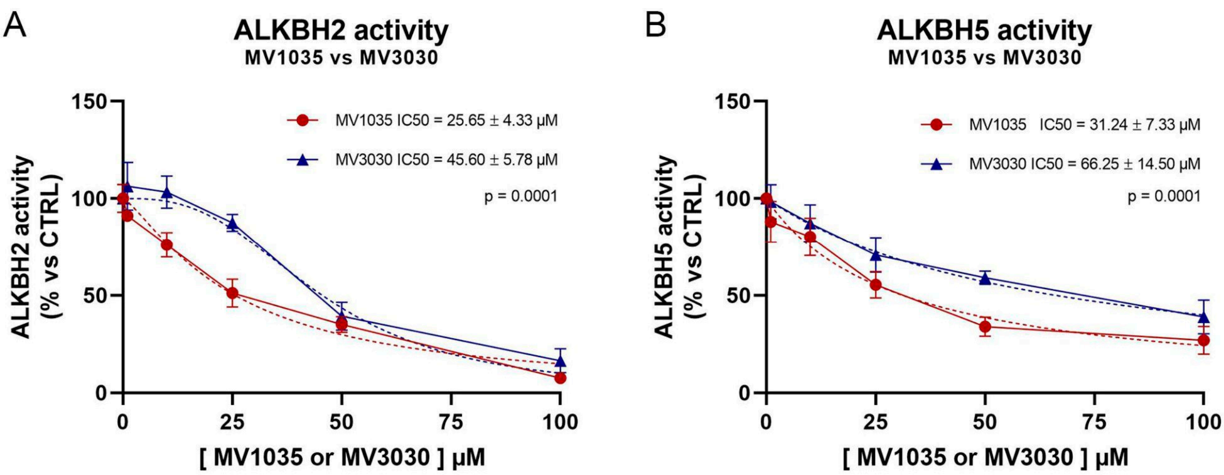

Supplementary Figure S10. Cell free ALKBH2 (A) and ALKBH5 (B) demethylase activity assay in presence of different concentrations of MV1035 or MV3030. Data are represented as mean percentage ± SD compared to untreated controls, arbitrarily set to 100 %. The Extra sum-of-squares F-test was used to calculate the *p*-value.

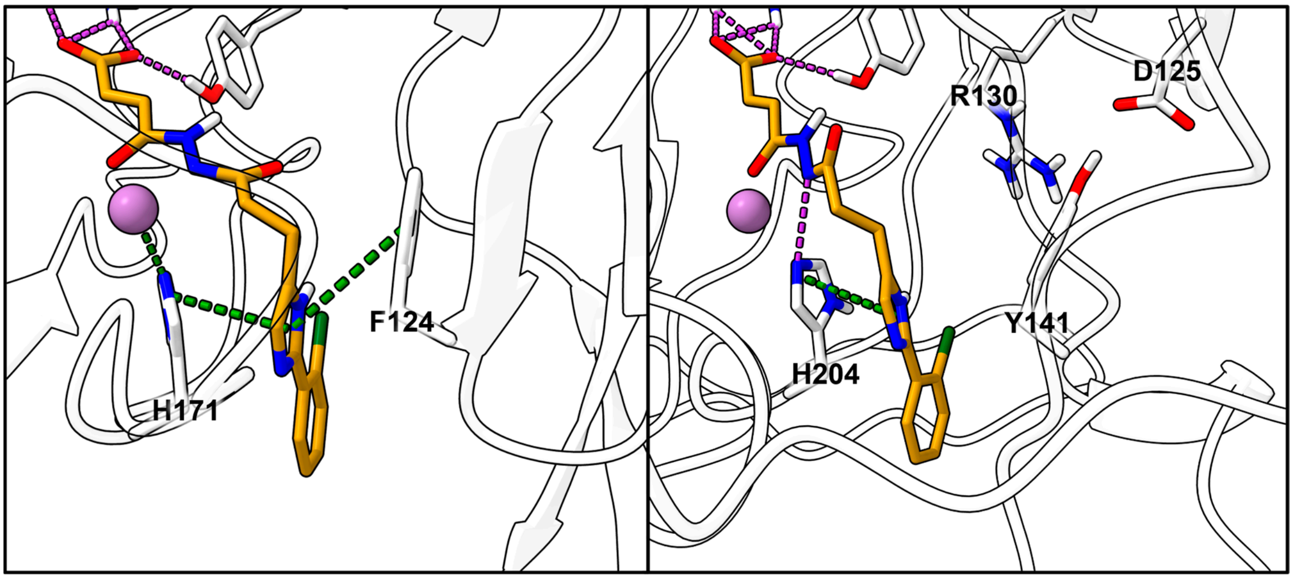

Supplementary Figure S11. Structural comparison of the predicted binding modes of the active fumarate hydrazide derivative within the ALKBH2 and ALKBH5 catalytic pockets. In the ALKBH2 active site (left), the phenyl-imidazole scaffold is optimally stabilized by π-π stacking interactions with residue Phe124. The ligand is deeply anchored by the distal fumarate hydrazide chain coordinating the catalytic metal ion (purple sphere). In the ALKBH5 active site (right), the corresponding residue Tyr141 exhibits a slightly shifted aromatic plane, influenced by its local environment and neighboring residues such as Arg130 and Asp125.

Supplementary Table S1. Predicted molecular docking scores for the initial set of compounds (MV1035, MV3003-MV3012, and 1-8) against ALKBH2 and ALKBH5 active sites.

Compound	Structure	ALKBH2 Docking Score (kcal/mol)	ALKBH5 Docking Score (kcal/mol)	Compound	Structure	ALKBH2 Docking Score (kcal/mol)	ALKBH5 Docking Score (kcal/mol)
MV1035	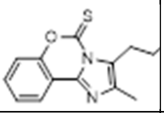	-5.1	-4.3	MV3012	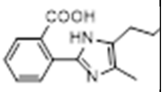	-8.7	-5.5
MV3003	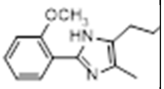	-4.8	-4.3	1	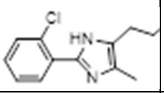	-4.8	-4.3
MV3004	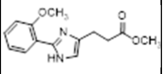	-5.8	-4.7	2	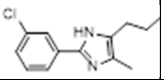	-4.5	-4.1
MV3005	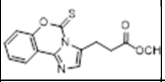	-5.6	-5.7	3	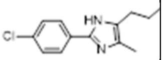	-4.9	-4.3
MV3006	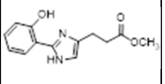	-5.4	-5.7	4	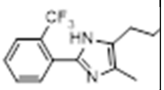	-4.6	-5.0
MV3007	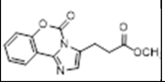	-5.5	-4.8	5	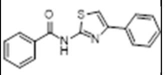	-5.7	-4.133
MV3009	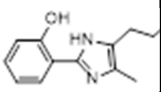	-4.8	-4.4	6	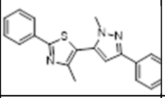	-5.6	-4.4
MV3010	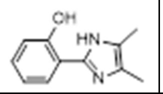	-3.9	-3.8	7	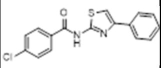	-5.8	-4.17
MV3011	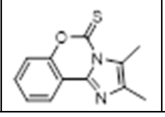	-5.0	-4.0	8	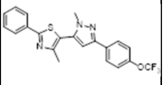	-5.7	-4.11

Supplementary Table S2. Authentication of the glioma stem cell (GSC) lines through short tandem repeat (STR) genotyping. The numerical value and the locus of each marker are reported. Regarding G179, in order to demonstrate the absence of cross-contamination and the reliability of the cell line, a comparison between the STR profiles of two different batches, the one used in Baronchelli's work (#) [1] and the one used in the current article (*), was performed. For GSC7, the STR profiles of the original tumor biopsy DNA (BP7) and the derived cell line were compared.

Marker	G179#	G179*	BP7	GSC7	Locus
Amelogenin	X,Y	X,Y	X,Y	X,Y	Xp22.1-p22.3 and Y
D3S1358	15.2,17	15.2,17	16,17	16,17	3p
D5S818	11	11	10,11	10,11	5q23.3-q32
D7S820	10	10	10	10	7q11.21-q22
D8S1179	8,15	8,15	14,17	14,17	8q24.13
D13S317	13,14	13,14	11,14	11,14	13q22-q31
D16S539	12	12	9	9	16q24.1
D18S51	15,16	15,16	11,15	11,15	18q21.3
D21S11	31.2,32.2	31.2,32.2	30,32.2	30,32.2	21q11-q21
FGA	23,26	23,26	22,26	22,26	4q28
PENTA D	11,12	11,12	9,13	9,13	21q
PENTA E	12	12	12,14	12,14	15q
THO1	7,9.3	7,9.3	6,8	6,8	11p15.5
TPOX	8	8	8	8	2p24-pter
vWA	16,17	16,17	16,17	16,17	12p13.31
CSF190	10,12	10,12	10,12	10,12	5q33.3-q34
